# The *D*^∗^*Dπ* and *B*^∗^*Bπ* couplings from light-cone sum rules

**DOI:** 10.1007/JHEP03(2021)016

**Published:** 2021-03-01

**Authors:** Alexander Khodjamirian, Blaženka Melić, Yu-Ming Wang, Yan-Bing Wei

**Affiliations:** 1 Theoretische Physik 1, Naturwissenschaftlich-Technische Fakultät, Universität Siegen, Walter-Flex-Straße 3, D-57068 Siegen, Germany; 2 Rudjer Boskovic Institute, Division of Theoretical Physics, Bijenička 54, HR-10000 Zagreb, Croatia; 3 School of Physics, Nankai University, Weijin Road 94, Tianjin, 300071 China; 4 Physik Department T31, Technische Universität München, James-Franck-Straße 1, D-85748 Garching, Germany

**Keywords:** NLO Computations, QCD Phenomenology

## Abstract

We revisit the calculation of the strong couplings *D*^∗^*Dπ* and *B*^∗^*Bπ* from the QCD light-cone sum rules using the pion light-cone distribution amplitudes. The accuracy of the correlation function, calculated from the operator product expansion near the light-cone, is upgraded by taking into account the gluon radiative corrections to the twist-3 terms. The double spectral density of the correlation function, including the twist-2, 3 terms at $\mathcal{O}\left({\alpha}_s\right)$ and the twist-4 LO terms, is presented in an analytical form for the first time. This form allows us to use various versions of the quark-hadron duality regions in the double dispersion relation underlying the sum rules. We predict ${g}_{D^{\ast } D\pi}={14.1}_{-1.2}^{+1.3}$ and ${g}_{B^{\ast } B\pi}={30.0}_{-2.4}^{+2.6}$ when the decay constants of heavy mesons entering the light-cone sum rule are taken from lattice QCD results. We compare our results with the experimental value for the charmed meson coupling and with the lattice QCD calculations.

ArXiv ePrint: 2011.11275

## Contents

**1 Introduction** **1**

**2 The LCSR method** **4**

**3 Double spectral density of the correlation function** **6**
- 3.1 Double spectral density at LO 6
- 3.2 Double spectral density at NLO 10

**4 Quark-hadron duality and the sum rule** **12**

**5 Numerical results** **16**

**6 Conclusions and perspectives** **29**

**A Pion light-cone DAs** **31**

**B Double spectral density at NLO** **33**

**C Two-point sum rules for heavy meson decay constants at NLO** **35**

## 1 Introduction

The strong couplings of heavy-light pseudoscalar and vector mesons with the pion belong to the most important hadronic parameters of heavy flavour physics. Our ability to calculate these couplings reflects the currently achieved progress in QCD and related effective theories. In the charm sector, the $D^*D\pi$ coupling has been measured, combining the branching fractions of the $D^* \to D\pi$ decays with the total width of $D^*$. The latter is currently available from the two experiments [1–3] and has a small error. The $B^*B\pi$ coupling cannot be directly measured, due to the lack of phase space for a $B^* \to B\pi$ decay. Still, this coupling is phenomenologically very important. It enters the residue of the $B^*$-meson pole in the vector $B \to \pi$ form factor used for the determination of the CKM parameter $V_{ub}$. Located very close to the kinematical threshold of the $B \to \pi\ell\bar{\nu}_\ell$ semileptonic transitions, the $B^*$ pole significantly influences the form factor at small hadronic recoil.

In the infinitely heavy-quark limit $m_b \to \infty$, the $B^*B\pi$ coupling turns into the "static" strong coupling of heavy-light mesons with the pion, a key parameter in the heavy-meson chiral perturbation theory (HM$\chi$ PT) [4–6]. There are several lattice QCD calculations of the heavy-meson strong couplings and their static limit, the most advanced ones, calculated with dynamical quarks, are in [7–12].

In [13], the $D^*D\pi$ and $B^*B\pi$ couplings have been calculated, employing the method of light-cone sum rules (LCSRs) in QCD [14–16]. The extension of LCSRs to strong couplings

JHEP03(2021)016

goes back to [17] where the pion-nucleon and $\rho\omega\pi$ couplings were calculated. The underlying object in this method is the vacuum-to-pion correlation function calculated near the light cone in terms of the operator product expansion (OPE) involving the universal pion light-cone distribution amplitudes (DAs) of growing twist. The same correlation function is used in the well established LCSRs for the $B \to \pi$ and $D \to \pi$ form factors, see e.g., [13, 18–22]. Importantly, the calculation of the $D^*D\pi$ and $B^*B\pi$ couplings is performed at a finite heavy-quark mass. Hence, not only the infinitely heavy quark limit of these couplings can be taken, but also the inverse mass corrections are accessible. The LCSR is obtained, employing analyticity in the two external momenta squared and matching the resulting double dispersion relation to the OPE result. The further steps follow the standard QCD sum rule technique and involve the quark-hadron duality approximation and the double Borel transformation. Due to the approximate degeneracy of vector and pseudoscalar heavy-light mesons (becoming exact in the infinitely heavy quark limit), equal Borel parameters are taken in both channels of the double dispersion relation. As a result, the LCSR predictions [13] for the $D^*D\pi$ and $B^*B\pi$ strong couplings at the leading order (LO) in $\alpha_s$ are sensitive to the values of the pion DAs at $u = \bar{u} = 1/2$ where $u$ and $\bar{u} \equiv 1 - u$ are the fractions of the pion momentum carried by the collinear quark and antiquark in the two-parton state of the pion. The shape of the pion twist-2 DA is usually described by an expansion in Gegenbauer polynomials based on the conformal partial-wave expansion. The value of this DA at the middle point provides a nontrivial constraint on the polynomial coefficients (Gegenbauer moments). Thus, LCSR for the strong coupling complements the information on the first few Gegenbauer moments available from other sources (e.g., lattice QCD calculation of the second moment and LCSRs for the pion form factors). Assessing the accuracy of the LCSR for the heavy-light strong couplings, one has to mention that the use of the double dispersion relation makes this sum rule more sensitive to the quark-hadron duality approximation than the LCSR for heavy-to-light form factors based on the single-variable dispersion relation. On the other hand, the accuracy of OPE in both sum rules is the same.

The interval for $g_{D^*D\pi}$ obtained in [13] appeared to be below the measured value by about 30%. The heavy-quark limit of this coupling obtained from LCSR was also smaller than the results of lattice QCD calculations. The gluon radiative correction to the twist-2 term of LCSR calculated in [23] did not remove this discrepancy. However, one should mention that theoretical uncertainties quoted in the previous analyses [13, 23] are incomplete and include only a part of parametrical uncertainties. In particular, the perturbative correction to the twist-3 term was not taken into account. The dependence on the form of duality region was also not completely investigated, moreover, the subleading twist-3, 4 contributions were included without the duality subtraction at tree level. Another critical point is the choice of decay constants of pseudoscalar and vector heavy-light mesons which multiply the strong coupling in LCSR, making the final result very sensitive to the values of these hadronic parameters.

A possibility to explain the deficit of the LCSR prediction for the heavy-light strong coupling is to allow for large contributions of excited heavy-light states to the double dispersion relation, as pointed out in [24] and discussed in more detail in [25]. Note however that this conjecture introduces an almost uncontrollable model-dependence in the

JHEP03(2021)016

hadronic part of the sum rule and leaves open the most important question: is there a duality region which effectively corresponds only to the ground-state contribution to the LCSR? Taking into account all above mentioned open aspects, it is timely to revisit the LCSR calculation of the strong couplings $B^*B\pi$ and $D^*D\pi$, upgrading and updating the earlier analyses in [13, 23].

In this paper we pursue three main goals. The first one is to improve the accuracy of the OPE for the underlying correlation function. To this end, we will include the next-to-leading-order (NLO) twist-3 term, calculating the corresponding gluon radiative corrections. We remind that in the LCSRs for the strong couplings the twist-3 part is comparable to the twist-2 part, their ratio being of $O(\mu_\pi/m_Q)$, where the chirally enhanced parameter $\mu_\pi = m_\pi^2/(m_u + m_d)$ is comparable with the heavy quark mass $m_Q = m_{c,b}$. Hence, by adding the gluon radiative correction to the twist-3 term, we will achieve the same NLO accuracy for both equally important parts of the OPE. Furthermore, we will, for the first time, represent both NLO corrections in a form of double dispersion relation with compact analytical expressions for the double spectral density.

Due to the importance of the twist-3 part, the LCSR considered here involves a double hierarchy of even (2, 4, 6,. . . ) and odd (3, 5,. . . ) twist terms. The twist-4 contributions known from previous analyses will be added in LO, which is sufficiently accurate since the twist-4 part is small with respect to the twist-2 LO part. Moreover, the twist-5, 6 contributions to the underlying correlation function calculated recently [26] in the factorizable approximation were found negligible. Also the next-to-next-to-leading-order (NNLO) correction to the twist-2 part obtained in [27] (in the large $\beta_0$ approximation) is very small. All this ensures that the OPE adopted here, including the twist-2, 3 terms at NLO, and the twist-4 term at LO, is sufficiently accurate.

Our second goal in this work is to update the input parameters in LCSR. In particular, in this paper we employ the $\overline{\text{MS}}$ mass scheme for the highly off-shell heavy quarks in the correlation function, which is a more appropriate choice than the pole-mass scheme employed in the earlier calculation. We also use the latest knowledge on the input parameters of pion DA's. For the decay constants of the vector and pseudoscalar heavy-light mesons we use the QCD two-point sum rules with the same NLO accuracy as LCSRs, employing the results of the updated analysis in [28] as well as the recent lattice QCD results. A more complete analysis of parametrical uncertainties of the sum rule results is done.

Finally, our third goal is to extend the quark-hadron duality approximation for the continuum subtraction to all twist-3 and 4 terms, in order to improve the procedure of subtraction of excited states in LCSR which was incomplete in [13]. The sensitivity of LCSRs to the form of the quark-hadron duality region in the double dispersion relation will be investigated.

The plan of this paper is as follows. After outlining the LCSR method in section 2, we present in section 3 the double spectral density of the correlation function in updated form, including the new twist-3 radiative correction. In section 4 we discuss different forms of the quark-hadron duality ansatz for the double dispersion relation. Section 5 contains the numerical analysis and section 6 is devoted to the concluding discussion. We present in appendix A necessary details on the pion DAs, in appendix B the expressions for the

JHEP03(2021)016

double spectral densities at NLO and in appendix C the sum rules for the heavy-light meson decay constants.

## 2 The LCSR method

Hereafter we use a generic notation $H^{(*)}$ for both pseudoscalar (vector) mesons $D^{(*)}$ and $B^{(*)}$. The strong $H^*H\pi$ coupling $g_{H^*H\pi}$ is defined as the invariant constant parametrizing the hadronic matrix element

$$\langle H^*(q)\pi(p)|H(p+q)\rangle = -g_{H^*H\pi}\, p^\mu \epsilon^{(H^*)}_\mu\,, \tag{2.1}$$

where the vector and pseudoscalar meson have four-momenta $q$ and $p+q$, respectively, and $\epsilon^{(H^*)}_\mu$ is the polarization vector of $H^*$. The infinitely heavy quark limit of the strong coupling:

$$\lim_{m_Q\to\infty} g_{H^*H\pi}/(2m_H) = \hat{g}/f_\pi\,, \tag{2.2}$$

where $f_\pi$ is the pion decay constant, determines the static coupling $\hat{g}$ that does not depend on the heavy mass scale and enters the HM$\chi$PT Lagrangian.

In [13] it was suggested to calculate the strong couplings (2.1) employing the LCSR based on the light-cone OPE for the vacuum-to-pion correlation function:

$$F_\mu(q,p) = i\int d^4x e^{iqx}\langle \pi(p)|T\{j_\mu(x), j_5(0)\}|0\rangle = F(q^2,(p+q)^2)\, p_\mu + \dots\,, \tag{2.3}$$

where $j_\mu = \bar{q}_1\gamma_\mu Q$ and $j_5 = (m_Q + m_{q_2})\bar{Q}\, i\gamma_5 q_2$ are the interpolating currents for the $H^*$ and $H$ mesons, respectively. In the above, $Q$ is a generic notation for the heavy quarks $c$ and $b$, and $q_{1,2}$ stand for the light quarks $u$ or $d$. The decay constants of heavy-light mesons needed here are defined as:

$$\langle 0|j_\mu|H^*(q)\rangle = m_{H^*}\epsilon^{(H^*)}_\mu f_{H^*}, \quad \langle 0|j_5|H(p+q)\rangle = m_H^2 f_H\,. \tag{2.4}$$

In (2.3) the relevant invariant amplitude $F$ multiplying $p_\mu$ is singled out, and the second Lorentz structure proportional to $q_\mu$ is indicated by ellipses. The pion is on shell and in what follows we adopt the chiral symmetry, putting $p^2 = m_\pi^2 = 0$ and neglecting the $u,d$ quark masses in the correlation function, adopting also the isospin symmetry. Note that the enhanced parameter

$$\mu_\pi = \frac{m_\pi^2}{m_u + m_d} \tag{2.5}$$

is retained in the chiral limit, since $m_\pi^2 \sim O(m_u + m_d)$. For the finite heavy quark mass in (2.3) we employ the $\overline{\text{MS}}$ scheme.

To derive LCSR for the strong coupling, following [13] one inserts the complete set of intermediate states with $H$ and $H^*$ quantum numbers in (2.3) and employs the double dispersion relation[1] for the amplitude $F(q^2,(p+q)^2)$ in the two independent variables $q^2$

[1]Double dispersion relations were used for QCD sum rules based on the local OPE, starting from [29] where the sum rules for charmonium radiative transitions were obtained. Another important application of double sum rules is the pion form factor [30, 31]; for the others see, e.g., the review [32]. The first application of LCSRs for hadronic couplings are presented in [14–17].

JHEP03(2021)016

and $(p+q)^2$:

$$F(q^2,(p+q)^2) = \frac{m_H^2 m_{H^*} f_H f_{H^*} g_{H^*H\pi}}{(m_H^2-(p+q)^2)(m_{H^*}^2-q^2)} + \iint\limits_{\Sigma} ds_2 ds_1 \frac{\rho^h(s_1,s_2)}{(s_2-(p+q)^2)(s_1-q^2)} + \dots\,, \tag{2.6}$$

where the possible subtraction terms are not shown. The latter include, in general, single dispersion integrals in the first variable $(p+q)^2$ combined with polynomials in the second variable $q^2$ and vice versa. All subtraction terms vanish after the double Borel transformation which will be applied to the relation (2.6).

The ground-state double-pole term in the above relation contains the product of $H^*H\pi$ strong coupling and decay constants. We denote by $\Sigma$ the two-dimensional region with the lower boundary $\{s_1 \geq (m_H+m_\pi)^2;\ s_2 \geq (m_{H^*}+m_\pi)^2\}$, where the hadronic spectral density of the continuum and excited states (with the $H^*$ and $H$ quantum numbers, respectively) denoted as $\rho^h(s_1,s_2)$ contributes.

At $q^2,(p+q)^2 \ll m_Q^2$, the dispersion relation (2.6) is matched to the result of the QCD calculation of $F(q^2,(p+q)^2)$. For the latter, we use the light-cone OPE in terms of pion DAs, and employ the most complete and up-to-date calculation in [21] that was used to obtain the LCSR for the $B\to\pi$ form factor. (see also [20], where, however the complete analytical expressions are not presented). Following the general outline of QCD sum rule derivation [33], we employ the quark-hadron duality ansatz. To this end, we will represent the OPE result for the correlation function in a form of double dispersion integral:

$$F^{(\mathrm{OPE})}(q^2,(p+q)^2) = \int\limits_{-\infty}^{\infty} \frac{ds_2}{s_2-(p+q)^2} \int\limits_{-\infty}^{\infty} \frac{ds_1}{s_1-q^2}\, \rho^{(\mathrm{OPE})}(s_1,s_2)\,, \tag{2.7}$$

with the double spectral density

$$\rho^{(\mathrm{OPE})}(s_1,s_2) \equiv \frac{1}{\pi^2}\mathrm{Im}_{s_1}\mathrm{Im}_{s_2} F^{(\mathrm{OPE})}(s_1,s_2)\,, \tag{2.8}$$

to be derived in the next section. Hereafter, we denote the variables $q^2$ and $(p+q)^2$ continued to their timelike regions as $s_1$ and $s_2$, respectively. For the sake of compactness, the lower limits of integration in (2.7) corresponding to the thresholds $s_{1,2} = m_Q^2$ are formally included in the spectral densities in a form of step functions and their derivatives. We also omit in (2.7) all subtraction terms that vanish after double Borel transformation.

Adopting the quark-hadron duality, we assume that the integral of the hadronic spectral density $\rho^h(s_1,s_2)$ taken over the two-dimensional region $\Sigma$ in (2.6) is equal to the integral of the OPE spectral density (2.8) taken over a certain region $\Sigma_0$ in the $(s_1,s_2)$ plane

$$\iint\limits_{\Sigma} ds_2\, ds_1 \frac{\rho^h(s_1,s_2)}{(s_2-(p+q)^2)(s_1-q^2)} = \iint\limits_{\Sigma_0} ds_2\, ds_1 \frac{\rho^{(\mathrm{OPE})}(s_1,s_2)}{(s_2-(p+q)^2)(s_1-q^2)}\,. \tag{2.9}$$

To proceed, we equate the double dispersion representations (2.6) and (2.7), substitute (2.9) to (2.6) and subtract the equal integrals over the region $\Sigma_0$ from both sides of this

JHEP03(2021)016

equation. For the remaining region dual to the ground-state contribution of the $H^* \to H\pi$ transition to (2.6) we introduce a generic notation:

$$\iint^{\Sigma_0} ds_2\, ds_1 \ldots = \int\limits_{-\infty}^{\infty} ds_2 \int\limits_{-\infty}^{\infty} ds_1 \ldots - \iint_{\Sigma_0} ds_2\, ds_1 \ldots . \tag{2.10}$$

The actual choice of this duality region will be discussed below. As a next step, we perform the double Borel transformation, defined as

$$\begin{aligned} f(M_1^2, M_2^2) = &\left[ \lim_{\substack{\{-q^2,\, n\}\to\infty, \\ -q^2/n = M_1^2}} \frac{(-q^2)^{n+1}}{n!} \left(\frac{d}{dq^2}\right)^n \right] \\ &\times \left[ \lim_{\substack{\{-(p+q)^2,\, k\}\to\infty, \\ -(p+q)^2/k = M_2^2}} \frac{(-(p+q)^2)^{k+1}}{k!} \left(\frac{d}{d(p+q)^2}\right)^k \right] f(q^2, (p+q)^2)\,. \end{aligned} \tag{2.11}$$

This transformation removes the subtraction terms and suppresses the higher-state contributions. The resulting LCSR for the product of the strong coupling and decay constants then reads

$$\begin{aligned} f_H f_{H^*} g_{H^*H\pi} = &\frac{1}{m_H^2 m_{H^*}} \exp\left(\frac{m_H^2}{M_2^2} + \frac{m_{H^*}^2}{M_1^2}\right) \\ &\times \iint^{\Sigma_0} ds_2\, ds_1\, \exp\left(-\frac{s_2}{M_2^2} - \frac{s_1}{M_1^2}\right) \rho^{(\mathrm{OPE})}(s_1, s_2)\,. \end{aligned} \tag{2.12}$$

The above sum rule yields the desired $H^*H\pi$ strong coupling, after dividing out the decay constants of $H^*$ and $H$. For the latter we will use the two-point QCD sum rules with the same NLO accuracy and the recent lattice QCD results.

## 3 Double spectral density of the correlation function

In this section, we derive the double spectral density $\rho^{(\mathrm{OPE})}(s_1, s_2)$ of the correlation function (2.3) calculated from the light-cone OPE. We will use the results presented in detail in [21]. The procedure to obtain the double spectral density was originally used in [13] at LO, including the twist-2, 3, 4 contributions. In [23], the NLO, $O(\alpha_s)$ correction to the twist-2 contribution was added to the double spectral density. The result was deduced from the NLO correction to the twist-2 term of the correlation function obtained in [18] (see also [19]). The new element to be included in our calculation is the NLO correction to the twist-3 part of $\rho^{(\mathrm{OPE})}(s_1, s_2)$. Apart from that, here we derive the double spectral density at LO in a more universal form, valid for any polynomial structure of the pion DA. We also use the updated nomenclature of the pion twist-4 DAs which differs from the one in [13].

### 3.1 Double spectral density at LO

The OPE near the light-cone $x^2 \sim 0$ for the correlation function (2.3) is valid if both external momenta squared $q^2$ and $(p+q)^2$ are far below the heavy quark threshold $m_Q^2$.

JHEP03(2021)016

More specifically, to warrant the power counting in the OPE, it is sufficient that

$$m_Q^2 - q^2 \sim m_Q^2 - (p+q)^2 \sim \mathcal{O}(m_Q \tau)\,, \tag{3.1}$$

where $\tau \gg \Lambda_{QCD}$ does not scale with $m_Q$. The heavy quark propagating in the correlation function is then highly virtual. The initial expression (2.3) is transformed into

$$F_\mu(q^2,(p+q)^2) = -im_Q \int d^4x e^{iqx} \langle \pi(p)|\bar{q}_1(x)\gamma_\mu S_Q(x,0)\gamma_5 q_2(0)|0\rangle\,, \tag{3.2}$$

where the heavy quark propagator $S_Q(x,0) = -i\,\langle 0|T\{Q(x),\,\bar{Q}(0)\}|0\rangle$ is expanded near the light-cone. In the adopted approximation, $S_Q(x,0)$ consists of the free-quark propagator and one-gluon emission term. In the correlation function (3.2) with the free heavy-quark propagator we encounter the vacuum-to-pion matrix element of the bilocal quark-antiquark operator $\bar{q}_1(x)\ldots q_2(0)$. In its turn, the gluon component of the propagator $S_Q(x,0)$ generates the contributions of the quark-antiquark-gluon operators $\bar{q}_1(x)\ldots G_{\mu\nu}(vx)\ldots q_2(0)$ with $0 \leq v \leq 1$. The emerging vacuum-to-pion matrix elements are expanded in terms of the pion quark-antiquark (quark-antiquark-gluon) DAs of growing twist $t = 2,3,4$ ($t = 3,4$), respectively. For the leading twist-2 DA we use the well-known standard definition:

$$\langle \pi^+(p)|\bar{u}(x)\gamma_\mu\gamma_5 d(0)|0\rangle = -if_\pi\, p_\mu \int\limits_0^1 du\, e^{iup\cdot x}\varphi_\pi(u)\,, \tag{3.3}$$

where the gauge link has been suppressed for brevity. All other pion light-cone DAs involved in the expressions presented below are defined e.g. in [21]. With the adopted twist-4 accuracy the resulting LO expression [21] for the invariant amplitude in (3.2) represents a sum of the separate twist and multiplicity contributions:

$$\begin{aligned} F^{(\mathrm{LO})}(q^2,(p+q)^2) = \big[&F^{(\mathrm{tw2,LO})} + F^{(\mathrm{tw3}p,\mathrm{LO})} + F^{(\mathrm{tw3}\sigma,\mathrm{LO})} \\ &+ F^{(\mathrm{tw3},\bar{q}Gq)} + F^{(\mathrm{tw4},\psi)} + F^{(\mathrm{tw4},\phi)} + F^{(\mathrm{tw4},\bar{q}Gq)}\big](q^2,(p+q)^2)\,, \end{aligned} \tag{3.4}$$

where the twist-2 contribution is

$$F^{(\mathrm{tw2,LO})}(q^2,(p+q)^2) = f_\pi m_Q^2 \int\limits_0^1 \frac{du}{m_Q^2-(q+up)^2}\,\varphi_\pi(u)\,, \tag{3.5}$$

and the two contributions of the pion two-particle twist-3 DAs are

$$F^{(\mathrm{tw3}p,\mathrm{LO})}(q^2,(p+q)^2) = f_\pi \mu_\pi m_Q \int\limits_0^1 \frac{du}{m_Q^2-(q+up)^2}\, u\,\phi^p_{3\pi}(u)\,, \tag{3.6}$$

and

$$F^{(\mathrm{tw3}\sigma,\mathrm{LO})}(q^2,(p+q)^2) = \frac{f_\pi\mu_\pi}{6} m_Q \int\limits_0^1 \frac{du}{m_Q^2-(q+up)^2}\left(2+\frac{m_Q^2+q^2}{m_Q^2-(q+up)^2}\right)\phi^\sigma_{3\pi}(u)\,. \tag{3.7}$$

JHEP03(2021)016

The second line in (3.4) contains subleading contribution of the twist-3 quark-antiquark-gluon DA:

$$F^{(\mathrm{tw3},\bar{q}Gq)}(q^2,(p{+}q)^2) = -4f_{3\pi}m_Q\int\limits_0^1 \frac{du}{m_Q^2-(q+up)^2}\left(1{-}\frac{m_Q^2-q^2}{m_Q^2-(q+up)^2}\right)\overline{\Phi}_{3\pi}(u)\,, \quad (3.8)$$

where we transformed the expression presented in [21] into a compact form, denoting the integrated three-particle DA as $\overline{\Phi}_{3\pi}(u)$. The remaining terms in (3.4) contain the twist-4 quark-antiquark DAs:

$$F^{(\mathrm{tw4},\psi)}(q^2,(p+q)^2) = -f_\pi m_Q^2\int\limits_0^1 \frac{du}{(m_Q^2-(q+up)^2)^2}\,\bar{\psi}_{4\pi}(u)\,, \quad (3.9)$$

$$F^{(\mathrm{tw4},\phi)}(q^2,(p+q)^2) = -f_\pi m_Q^4\int\limits_0^1 \frac{du}{2(m_Q^2-(q+up)^2)^3}\,\phi_{4\pi}(u)\,, \quad (3.10)$$

and the integrated linear combinations of the twist-4 quark-antiquark-gluon DAs,

$$F^{(\mathrm{tw4},\bar{q}Gq)}(q^2,(p+q)^2) = f_\pi m_Q^2\int\limits_0^1 \frac{du}{(m_Q^2-(q+up)^2)^2}\,\overline{\Phi}_{4\pi}(u)\,. \quad (3.11)$$

The expressions for all pion DAs and their combinations entering (3.5)–(3.11) are given in appendix A. We use the same updated set of twist-3 and 4 DAs from [34] as in [21]. Their definitions go back to the original work in [35]. The form of each DA follows from the conformal partial-wave expansion and is given by a combination of certain orthogonal polynomials in the momentum variables, such as the variable $u$ in (3.3). The input parameters in DAs include the overall normalization factors, e.g., $f_\pi$ in (3.3), and the coefficients at the polynomials normalized at a certain default normalization scale.

Our task is to derive a double dispersion relation in the form (2.7) for each separate term in the OPE (3.4). To this end, we notice that all contributions of the three-particle DAs in (3.4) have the form of a convolution integral of a single variable $u$, similar to the contributions of the two-particle DAs. Moreover, all expressions in (3.5)–(3.11) are reduced to linear combinations of the two generic integrals

$$F_\ell^{(\phi)}(q^2,(p+q)^2) \equiv \int\limits_0^1 du\frac{\phi(u)}{\left[m_Q^2-\bar{u}q^2-u(p+q)^2\right]^\ell}\,,$$

$$\widetilde{F}_\ell^{(\phi)}(q^2,(p+q)^2) \equiv \int\limits_0^1 du\,\frac{q^2\phi(u)}{\left[m_Q^2-\bar{u}q^2-u(p+q)^2\right]^\ell}\,, \quad (3.12)$$

where $\ell = 1,2,3$ and $\phi(u)$ has to be replaced by a respective DA, entering (3.5)–(3.11):

$$\phi = \{\varphi_\pi, u\phi^p_{3\pi}, \phi^\sigma_{3\pi}, \overline{\Phi}_{3\pi}, \bar{\psi}_{4\pi}, \phi_{4\pi}, \overline{\Phi}_{4\pi}\}.$$

Note that in (3.12) we have transformed the denominator, making use of

$$m_Q^2-(q+up)^2 = m_Q^2-\bar{u}q^2-u(p+q)^2,$$

valid at $p^2 = 0$ i.e. in a massless pion approximation utilized throughout the paper. Furthermore, since we aim at the most general form of the double dispersion relation, it is convenient to perform a Taylor expansion of all pion DAs or their integrated combinations entering (3.5)–(3.11)

$$\phi(u) = \sum_{k=0}^{\infty} c_k^{(\phi)}\, u^k\,. \tag{3.13}$$

The expansion (3.13) is convergent for all DAs including the twist-4 two-particle DA $\phi_{4\pi}(u)$ in (3.10), which contains logarithmic terms of the type $u^k \ln u$ and $\bar{u}^k \ln \bar{u}$ with $k \geq 3$.

Consequently, it is sufficient to find the double spectral representation for the first integral in (3.12) in which $\phi(u)$ is replaced by the power $u^k$:

$$\int_0^1 du \frac{u^k}{\left[m_Q^2 - \bar{u}q^2 - u(p+q)^2\right]^\ell} = \int \frac{ds_2}{s_2-(p+q)^2} \int \frac{ds_1}{s_1-q^2}\, \rho_{\ell k}(s_1,s_2)\,, \tag{3.14}$$

at arbitrary $\ell \geq 1$ and $k > 0$, so that the second integral in (3.12) is obtained by a simple replacement of $\rho_{\ell k}(s_1,s_2)$ with $\tilde{\rho}_{\ell k}(s_1,s_2)$ where

$$\widetilde{\rho}_{\ell k}(s_1,s_2) = s_1 \rho_{\ell k}(s_1,s_2)\,. \tag{3.15}$$

As already said before, we hereafter neglect the typical subtraction terms which vanish after the double Borel transformation. The formula for the spectral density $\rho_{\ell k}$ can be directly taken from the recent work [36] where it was derived in a different context (see also [37] for an alternative technique suitable for the analogous problems but with the nonvanishing light-hadron mass). We have:

$$\rho_{\ell k}(s_1,s_2) = \frac{(-1)^{\ell-1}(-1)^k}{(\ell-1)!\,k!} \frac{d^{\,\ell-1}}{{dm_Q^2}^{\,\ell-1}} \left[ \left(s_1 - m_Q^2\right)^k \theta(s_2 - m_Q^2) \right] \delta^{(k)}(s_1 - s_2)\,, \tag{3.16}$$

where $\delta^{(k)}(x) \equiv d^k/dx^k[\delta(x)]$. Note that at $\ell = 1$ the expression for $\rho_{1k}(s_1,s_2)$ coincides with the one used in [13].

The integrals in (3.12) containing a generic DA $\phi(u)$ can be written as

$$F_\ell^{(\phi)}(q^2,(p+q)^2) = \int \frac{ds_2}{s_2-(p+q)^2} \int \frac{ds_1}{s_1-q^2}\, \rho_\ell^{(\phi)}(s_1,s_2)\,, \tag{3.17}$$

and the analogous representation for $\widetilde{F}_\ell^{(\phi)}$ with $\widetilde{\rho}_\ell^{(\phi)}(s_1,s_2)$, where the cumulative spectral densities are obtained combining the expansion (3.13) with the "elementary" spectral densities (3.16),

$$\rho_\ell^{(\phi)}(s_1,s_2) = \sum_{k=0}^{\infty} c_k^{(\phi)} \rho_{\ell k}(s_1,s_2), \qquad \widetilde{\rho}_\ell^{(\phi)}(s_1,s_2) = \sum_{k=0}^{\infty} c_k^{(\phi)} \widetilde{\rho}_{\ell k}(s_1,s_2). \tag{3.18}$$

Replacing one by one all twist and multiplicity components in the sum (3.4) by their double dispersion forms, we obtain the double spectral density for the LO part of the correlation

JHEP03(2021)016

function

$$\rho^{(\mathrm{LO})}(s_1,s_2) = f_\pi\, m_Q^2\Bigg[\rho_1^{(\varphi_\pi)} + \frac{\mu_\pi}{m_Q}\left(\rho_1^{(u\phi^p_{3\pi})} + \frac{1}{3}\,\rho_1^{(\phi^\sigma_{3\pi})} + \frac{m_Q^2}{6}\,\rho_2^{(\phi^\sigma_{3\pi})} + \frac{1}{6}\tilde{\rho}_2^{(\phi^\sigma_{3\pi})}\right) \tag{3.19}$$
$$+\,4\,\frac{f_{3\pi}}{f_\pi\, m_Q}\left(-\rho_1^{(\bar\Phi_{3\pi})} + m_Q^2\,\rho_2^{(\bar\Phi_{3\pi})} - \tilde{\rho}_2^{(\bar\Phi_{3\pi})}\right) - \rho_2^{(\bar\psi_{4\pi})} - \frac{m_Q^2}{2}\,\rho_3^{(\phi_{4\pi})} + \rho_2^{(\bar\Phi_{4\pi})}\Bigg](s_1,s_2)\,,$$

where each term has a form of expansion (3.18) with the coefficients $c_k^{(\phi)}$ easily determined from the polynomial form of the DAs explicitly presented in appendix A. The expression (3.19) is new. Note that it is valid in the chiral limit, i.e. at $p^2 = m_\pi^2 = 0$. To give useful examples, we present the contribution to $\rho^{(\mathrm{LO})}$ of the twist-2 and twist-3 DAs taken in the asymptotic form:

$$\begin{aligned}
\rho_1^{(\varphi_\pi)}(s_1,s_2) &= -6\left[(s_1-m_Q^2)\delta^{(1)}(s_1-s_2) + \frac{1}{2}(s_1-m_Q^2)^2\delta^{(2)}(s_1-s_2)\right]\,\theta(s_2-m_Q^2)\,,\\
\rho_1^{(u\phi^p_{3\pi})}(s_1,s_2) &= -(s_1-m_Q^2)\delta^{(1)}(s_1-s_2)\,\theta(s_2-m_Q^2)\,,\\
\rho_1^{(\phi^\sigma_{3\pi})}(s_1,s_2) &= \rho_1^{(\varphi_\pi)}(s_1,s_2)\,,\\
\rho_2^{(\phi^\sigma_{3\pi})}(s_1,s_2) &= -6\left[\delta^{(1)}(s_1-s_2) + (s_1-m_Q^2)\delta^{(2)}(s_1-s_2)\right]\theta(s_2-m_Q^2)\\
&\quad - 6\left[\delta^{(1)}(s_1-s_2) + \frac{1}{2}(s_1-m_Q^2)\delta^{(2)}(s_1-s_2)\right](s_1-m_Q^2)\delta(s_2-m_Q^2)\,,\\
\tilde{\rho}_2^{(\phi^\sigma_{3\pi})}(s_1,s_2) &= s_1\,\rho_2^{(\phi^\sigma_{3\pi})}(s_1,s_2)\,.
\end{aligned} \tag{3.20}$$

The expression (3.19) enables to write down the double spectral representation of $F^{(\mathrm{LO})}$ in a form (2.7) and to perform a double Borel transformation in a general case of the two unequal parameters $M_1^2, M_2^2$. In what follows we put $M_1 = M_2$ as motivated by the heavy quark symmetry. After integrating (3.19) over the duality region specified in the next subsection, we will see that the resulting LO part of the sum rule is substantially simplified and reduced to a linear combination of DAs or their derivatives at the middle point.

### 3.2 Double spectral density at NLO

To NLO accuracy, the invariant amplitude we are interested in the correlation function (2.3) becomes

$$F^{(\mathrm{OPE})}(q^2,(p+q)^2) = F^{(\mathrm{LO})}(q^2,(p+q)^2) + \frac{\alpha_s C_F}{4\pi}F^{(\mathrm{NLO})}(q^2,(p+q)^2)\,, \tag{3.21}$$

where the gluon radiative corrections at $O(\alpha_s)$ have been calculated in [18, 19] for the twist-2 part and in [21, 38] for the twist-3 part. The result of this calculation is cast in a form of the convolution of the hard-scattering amplitudes and the twist-2 and twist-3 DAs:

$$F^{(\mathrm{NLO})}(q^2,(p+q)^2) = f_\pi\int\limits_0^1 du\Big\{\varphi_\pi(u)\,T_1(q^2,(p+q)^2,u) \tag{3.22}$$
$$+\frac{\mu_\pi}{m_Q}\left[\phi^p_{3\pi}(u)T_1^p(q^2,(p+q)^2,u) + \phi^\sigma_{3\pi}(u)T_1^\sigma(q^2,(p+q)^2,u)\right]\Big\},$$

JHEP03(2021)016

where the expressions for the twist-2 amplitude $T_1$, and the twist-3 amplitudes $T_1^p$ and $T_1^\sigma$ can be found in [21]. Here we do not show explicitly the residual scale dependence of the hard-scattering amplitudes and of the nonasymptotic parts of pion DAs. Note that, as explained in [21], the twist-3 part of (3.22) is only applicable to the asymptotic DAs $\phi_{3\pi}^p(u)$ and $\phi_{3\pi}^\sigma(u)$, (obtained by putting in (A.4) the parameter $f_{3\pi} \to 0$) because the hard-scattering amplitudes $T_1^{p,\sigma}$ are determined perturbatively without taking into account the renormalization-mixing effects between the two- and three-particle DAs. Furthermore, in [21] the NLO part of the correlation function was represented in a form of a single-variable dispersion relation, calculating the imaginary part in $s_2$ which is the timelike continuation of the variable $(p+q)^2$:

$$\mathrm{Im}_{s_2} F^{(\mathrm{NLO})}(q^2, s_2) = \int\limits_0^1 du \Big\{ \varphi_\pi(u)\, \mathrm{Im}_{s_2} T_1(q^2, s_2, u, \mu) + \frac{\mu_\pi}{m_Q} \Big[ \phi_{3\pi}^p(u)\, \mathrm{Im}_{s_2} T_1^p(q^2, s_2, u, \mu) + \phi_{3\pi}^\sigma(u)\, \mathrm{Im}_{s_2} T_1^\sigma(q^2, s_2, u, \mu) \Big] \Big\}, \tag{3.23}$$

at fixed $q^2 < m_Q^2$. The above expression was used to derive the NLO terms in LCSRs for the $H \to \pi$ form factors.

Here we need to make a step further and obtain the double spectral density

$$\rho^{(\mathrm{NLO})}(s_1, s_2) \equiv \frac{1}{\pi^2} \mathrm{Im}_{s_1} \mathrm{Im}_{s_2} F^{(\mathrm{NLO})}(s_1, s_2)\,, \tag{3.24}$$

analytically continuing (3.23) in the variable $q^2 \to s_1$. This double density consists of the three contributions stemming from the twist-2 and twist-3 quark-antiquark DAs:

$$\rho^{(\mathrm{NLO})}(s_1, s_2) = \rho^{(\mathrm{tw2,NLO})}(s_1, s_2) + \rho^{(\mathrm{tw3}p,\mathrm{NLO})}(s_1, s_2) + \rho^{(\mathrm{tw3}\sigma,\mathrm{NLO})}(s_1, s_2)\,. \tag{3.25}$$

We will use the asymptotic DAs for all three NLO terms. To justify this approximation, we note that at LO the nonasymptotic contributions due to the Gegenbauer moments in the twist-2 DA (see (A.1)) contribute at the level of a few percent to LCSR, if a typical magnitude of the moments $a_2, a_4$ is taken (see the section on numerical results below). An additional $O(\alpha_s)$ factor will suppress these contributions well below the level of the parametric uncertainties of the sum rule. For the twist-3 part the nonasymptotic effects at NLO are even smaller, because already at LO these effects are determined by a combination of parameters $f_{3\pi}/(\mu_\pi f_\pi) \sim 0.01$.

For the asymptotic DAs, the calculation of $\rho^{(\mathrm{NLO})}(s_1, s_2)$ simplifies since the integral over $u$ in (3.23) is performed before analytically continuing the variable $q^2$ to $q^2 = s_1 > m_b^2$. The expressions for the imaginary parts in $(p+q)^2$ of the hard scattering amplitudes in (3.23) are taken from [21].

The twist-2 term in (3.25) was already calculated in [23]. We have recalculated it and confirm the expression presented there. The resulting expression for $\rho^{(\mathrm{tw2,NLO})}(s_1, s_2)$ is presented in the appendix B. Note that, since we are now using the $\overline{\mathrm{MS}}$ scheme for the heavy quark mass, an additional $O(\alpha_s)$ piece has to be added to the expression in [23] obtained for the pole mass of the heavy quark.

The derivation of the NLO twist-3 terms in the double spectral density (3.25) is new. In the course of calculation we found that the resulting expressions for $\rho^{(\mathrm{tw}3p,\mathrm{NLO})}(s, s_2)$ and $\rho^{(\mathrm{tw}3\sigma,\mathrm{NLO})}(s_1, s_2)$ contain terms which cancel each other. Therefore the final expression of the sum of the two denoted as $\rho^{(\mathrm{tw}3,\mathrm{NLO})}(s, s_2)$ is more compact. It is presented in appendix B.

## 4 Quark-hadron duality and the sum rule

Having calculated the double spectral density (2.8) as

$$\rho^{(\mathrm{OPE})}(s_1, s_2) = \rho^{(\mathrm{LO})}(s_1, s_2) + \frac{\alpha_s C_F}{4\pi}\rho^{(\mathrm{NLO})}(s_1, s_2)\,, \tag{4.1}$$

where the LO part is given in (3.19) and the NLO part represents the sum of the twist-2 and twist-3 parts given, respectively in (B.1) and (B.4), we are in a position to perform the integration over a duality region in the LCSR (2.12). In the $\{s_1, s_2\}$ plane, the lower boundary of that region is determined by the heavy quark threshold (in the chiral limit for light quarks) and is given by the straight lines $s_1 = m_Q^2$ and $s_2 = m_Q^2$. For the upper boundary symbolized by $\Sigma_0$ in (2.12) there is a multiple choice.

As argued in [39], the triangular-type duality region is preferable in the HQET sum rule for the Isgur-Wise function, based on the local OPE. This choice was also supported in [40] by invoking the double sum rules in nonrelativistic quantum mechanics. Here we follow the same guidelines in choosing the duality region, notwithstanding that the LCSR for the $H^*H\pi$ coupling is based on a different type of OPE, with an interplay of the collinear and soft QCD dynamics. In [40] it was shown that duality ansatz works only if the spectral densities are integrated first over the direction perpendicular to the diagonal $s_1 = s_2$ in the $s_{1,2}$ plane. Therefore, we only choose among the regions which process a smooth border crossing of the diagonal and allow for evaluating the obtained dispersion integrals properly, implying that the square duality region with a sharp corner on the diagonal has to be discarded as discussed below.

The working duality region includes an interval of the diagonal $s_1 = s_2$ with a length characterized by the effective threshold $s_0$, as illustrated in figure 1. The value of this parameter is expected in the ballpark of the duality threshold in the LCSRs for the $H \to \pi$ form factors. Our choice for the duality region is motivated by the fact that the dominant LO part of the spectral density (4.1) is concentrated near diagonal, since $\rho^{(\mathrm{LO})}(s_1, s_2)$ represents a sum of terms proportional to $\delta(s_1 - s_2)$ and its first few derivatives. Due to this property of the LO spectral density, the shape of the two-dimensional duality region becomes inessential. However, since we also include the $\rho^{(\mathrm{NLO})}(s_1, s_2)$ part, which contains nonvanishing terms at $s_1 \neq s_2$, a certain dependence on the adopted shape of the duality region will occur. In order to assess this effect in the NLO part, we will probe the duality regions with different shapes but possessing the same diagonal interval along the line $s_1 = s_2$. To this end, it is convenient to use the parameterization of the boundaries suggested in [15]:

$$\left(\frac{s_1}{s_*}\right)^{\alpha} + \left(\frac{s_2}{s_*}\right)^{\alpha} \leq 1\,, \qquad s_1,\, s_2 \geq m_Q^2\,. \tag{4.2}$$

JHEP03(2021)016

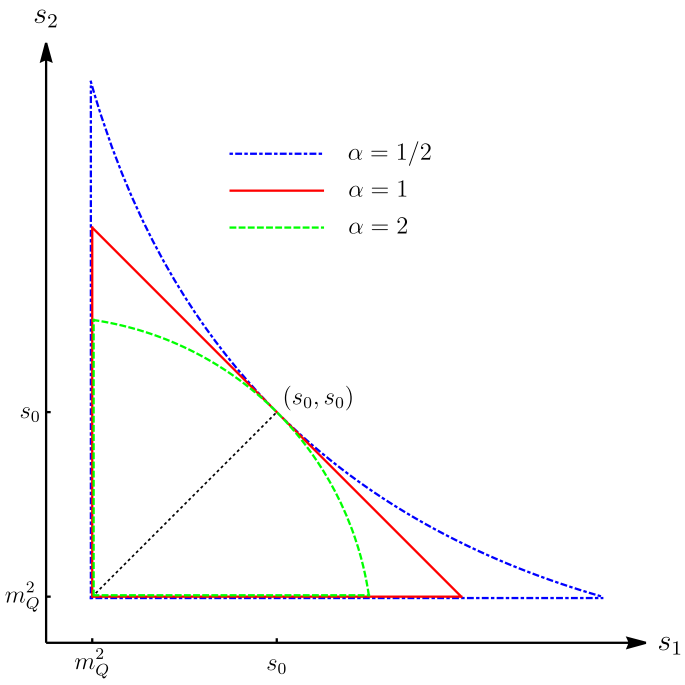

**Figure 1**. The duality regions defined in eq. (4.2).

We will probe the three regions, generated at

$$
\begin{aligned}
&\alpha = 1\,, && s_* = 2s_0\,, && \text{(triangle)};\\
&\alpha = \frac{1}{2}\,, && s_* = 4s_0\,, && \text{(concave)};\\
&\alpha = 2\,, && s_* = \sqrt{2}s_0\,, && \text{(convex)};
\end{aligned}
\tag{4.3}
$$

where $s_*$ is adjusted to provide equal diagonal intervals. These regions are shown, respectively, in figure 1.

Note that in the limit $\{\alpha \to \infty,\, s_* \to s_0\}$, the parameterization (4.2) represents a square with the side $s_0$. In this limiting case, the integration of both NLO twist-2 and twist-3 spectral densities (B.1) and (B.4) develops a spurious divergence at the vertex $s_1 = s_2 = s_0$ of the square. This divergence can be traced back to the presence of the terms involving

$$
\frac{d^3}{ds_1^3}\Big(\ln|s_1 - s_2|\Big)\,.
$$

To avoid such spurious divergences, it is sufficient to replace the outmost vertex of the square duality region with a smooth, infinitesimally small curve. It is clear that the terms in the NLO spectral density containing $\delta(s_1 - s_2)$ and its derivatives, after integration over any of the duality regions defined by (4.2) and (4.3) and shown in figure 1 yield equal contributions. Consequently, only those contributions at the NLO, eqs. (B.1) and (B.4), which do not contain the delta-function and its derivatives are sensitive to the choice of the duality region.

JHEP03(2021)016

Apart from this, presumably minor effect, which we will numerically study in the next section, the whole LO and the main part of NLO contributions originate from the integration over the interval on the diagonal which is equal for all duality regions.

Hence, we hereafter adopt the most convenient choice: the triangular region, satisfying the condition

$$s_1 + s_2 \leq 2\, s_0. \tag{4.4}$$

Returning to the LCSR (2.12), we subsequently assume equal Borel parameters

$$M_1^2 = M_2^2 = 2M^2$$

and rewrite the sum rule as

$$f_H f_{H^*}\, g_{H^*H\pi} = \frac{1}{m_H^2 m_{H^*}} \exp\left(\frac{m_H^2 + m_{H^*}^2}{2M^2}\right) \left[\mathcal{F}^{(\mathrm{LO})}(M^2, s_0) + \frac{\alpha_s C_F}{4\pi}\, \mathcal{F}^{(\mathrm{NLO})}(M^2, s_0)\right], \tag{4.5}$$

introducing the compact notation for the integrals over the triangular duality region,

$$\mathcal{F}^{(\mathrm{LO}),(\mathrm{NLO})}(M^2, s_0) \equiv \int\limits_{-\infty}^{\infty} ds_1 \int\limits_{-\infty}^{\infty} ds_2\, \theta(2s_0 - s_1 - s_2)\, \exp\left(-\frac{s_1 + s_2}{2\, M^2}\right) \rho^{(\mathrm{LO}),(\mathrm{NLO})}(s_1, s_2)\,. \tag{4.6}$$

where the lower limits determined by the heavy quark mass are implicitly given by the theta functions in the expressions of the spectral densities.

To calculate the LO part in (4.5), we use (3.19) where the spectral density $\rho^{(\mathrm{LO})}$ is expressed via contributions of the separate DAs. We then reduce $\mathcal{F}^{(\mathrm{LO})}(M^2, s_0)$ to a linear combination of the integrals:

$$\mathcal{F}_\ell^{(\phi)}(M^2, s_0) \equiv \int\limits_{-\infty}^{\infty} ds_1 \int\limits_{-\infty}^{\infty} ds_2\, \theta(2s_0 - s_1 - s_2)\, \exp\left(-\frac{s_1 + s_2}{2\, M^2}\right) \rho_\ell^{(\phi)}(s_1, s_2)\,, \tag{4.7}$$

where $\phi = \varphi_\pi, u\phi_{3\pi}^p, \phi_{3\pi}^\sigma$, etc. In addition, we define the similar integrals $\widetilde{\mathcal{F}}_\ell^{(\phi)}(M^2, s_0)$ over $\widetilde{\rho}_\ell^{(\phi)}$. It is now straightforward to replace each DA by its Taylor expansion (3.13) and expand the density $\rho_\ell^{(\phi)}$ in the elementary components according to (3.18). In fact, in the case of triangular duality region the resulting formulas for the integrals $\mathcal{F}_\ell^{(\phi)}$ and $\widetilde{\mathcal{F}}_\ell^{(\phi)}$ can be written in a universal form valid for a generic DA. To this end, following [13], we transform the integration variables in (4.7):

$$s_1 = s(1 - v)\,, \quad s_2 = sv \tag{4.8}$$

or, inversely

$$s = s_1 + s_2\,, \qquad v = \frac{s_2}{s_1 + s_2}\,,$$

so that $(s_1 - s_2) \to s(1 - 2v)$, allowing us to integrate out the $\delta(s_1 - s_2) = \delta(1 - 2v)/s$ functions and their derivatives over $v$. On the other hand, the exponential factor in (4.7)

becomes independent of $v$. As a result, the Taylor expansion of an arbitrary DA $\phi(u)$ reduces to its value or its derivative at $u = 1/2$ and we obtain

$$\begin{aligned}
&\mathcal{F}_\ell^{(\phi)}(M^2,s_0) \qquad\qquad (4.9)\\
&= \frac{(-1)^\ell}{(\ell-1)!}\Big\{(-1)^\ell (M^2)^{2-\ell}\exp\Big(-\frac{m_Q^2}{M^2}\Big) + \delta_{\ell 1} M^2 \exp\Big(-\frac{s_0}{M^2}\Big)\Big\}\,\phi(u)\Big|_{u=\frac{1}{2}}\,,\\
&\widetilde{\mathcal{F}}_\ell^{(\phi)}(M^2,s_0)\\
&= -\frac{(-1)^\ell}{2(\ell-1)!}\,\frac{d^{\ell-1}}{dm_Q^{2\,\ell-1}}\int_{2m_Q^2}^{2s_0} ds\,\exp\Big(-\frac{s}{2M^2}\Big)\left[u\left(\frac{s}{2}-m_Q^2\right)\phi'(u)+\frac{s}{2}\,\phi(u)\right]\Big|_{u=\frac{1}{2}}\,.
\end{aligned}$$

As we will see below, only $\widetilde{\mathcal{F}}_2$ contributes, hence for convenience we quote the second integral in (4.9) at $\ell = 2$

$$\widetilde{\mathcal{F}}_2^{(\phi)}(M^2,s_0) = m_Q^2\exp\Big(-\frac{m_Q^2}{M^2}\Big)\phi(u) + M^2\left[\exp\Big(-\frac{m_Q^2}{M^2}\Big)-\exp\Big(-\frac{s_0}{M^2}\Big)\right]u\,\phi'(u)\Big|_{u=\frac{1}{2}}\,. \tag{4.10}$$

The above formulas are also valid for the twist-4 DA $\phi_{4\pi}$ which contains the specific $u^k \ln u$ and $\bar{u}^k \ln \bar{u}$ terms with $k \geq 3$.

Finally, the LO part of the LCSR in (4.5) is obtained in a form of a linear combination of the separate DA contributions:

$$\begin{aligned}
\mathcal{F}^{(\mathrm{LO})}(M^2,s_0) = f_\pi m_Q^2\Big[&\mathcal{F}_1^{(\varphi_\pi)} + \frac{\mu_\pi}{m_Q}\mathcal{F}_1^{(u\phi_{3\pi}^p)} + \frac{1}{6}\frac{\mu_\pi}{m_Q}\Big(2\,\mathcal{F}_1^{(\phi_{3\pi}^\sigma)} + m_Q^2\,\mathcal{F}_2^{(\phi_{3\pi}^\sigma)} + \widetilde{\mathcal{F}}_2^{(\phi_{3\pi}^\sigma)}\Big)\\
&-4\frac{f_{3\pi}}{f_\pi m_Q}\Big(\mathcal{F}_1^{(\Phi_{3\pi})} - m_Q^2\,\mathcal{F}_2^{(\Phi_{3\pi})} + \widetilde{\mathcal{F}}_2^{(\Phi_{3\pi})}\Big) - \mathcal{F}_2^{(\bar\psi_{4\pi})} - \frac{m_Q^2}{2}\mathcal{F}_3^{(\phi_{4\pi})} + \mathcal{F}_2^{(\bar\Phi_{4\pi})}\Big](M^2,s_0)\,.
\end{aligned} \tag{4.11}$$

Using (4.9), we obtain a compact explicit expression which is straightforward to use in the numerical analysis of the LCSR (4.5):

$$\begin{aligned}
\mathcal{F}^{(\mathrm{LO})}(M^2,s_0) = f_\pi m_Q^2\Big\{&M^2\left[\exp\Big(-\frac{m_Q^2}{M^2}\Big)-\exp\Big(-\frac{s_0}{M^2}\Big)\right]\Big[\varphi_\pi(u)\\
&+\frac{\mu_\pi}{m_Q}\Big(u\,\phi_{3\pi}^p + \frac{1}{3}\phi_{3\pi}^\sigma + \frac{1}{6}u\frac{d\phi_{3\pi}^\sigma}{du}\Big)(u) - \frac{4f_{3\pi}}{f_\pi m_Q}\Big(\Phi_{3\pi} + u\frac{d\Phi_{3\pi}}{du}\Big)(u)\Big]\\
&+\exp\Big(-\frac{m_Q^2}{M^2}\Big)\left[\frac{\mu_\pi m_Q}{3}\phi_{3\pi}^\sigma - \bar\psi_{4\pi} - \frac{1}{4}\frac{m_Q^2}{M^2}\phi_{4\pi} + \bar\Phi_{4\pi}\right](u)\Big\}\Big|_{u=\frac{1}{2}}\,.
\end{aligned} \tag{4.12}$$

Comparing term by term this expression with the one obtained in [13], we found that they coincide, although no explicit duality subtraction was applied to the twist-4 terms in [13]. In fact, the peculiar feature of the latter terms is that at equal Borel parameters the $s_0$-dependent terms vanish, as one can realize using the expressions for the double spectral density derived here and valid for a generic Taylor-expandable DA.

It remains to obtain the NLO part of (4.5). We have to calculate $\mathcal{F}^{(\mathrm{NLO})}(M^2,s_0)$ defined in (4.6) by substituting the sum of the twist-2 and twist-3 NLO double spectral densities $\rho^{(\mathrm{tw2,NLO})}(s_1,s_2)$ and $\rho^{(\mathrm{tw3,NLO})}(s_1,s_2)$ presented in (B.1) and (B.4) of appendix B.

JHEP03(2021)016

The resulting expressions of $\mathcal{F}^{(\mathrm{NLO})}(M^2, s_0)$ for the triangular duality region reads:

$$\mathcal{F}^{(\mathrm{NLO})}(M^2, s_0) = f_\pi\, m_Q^2 \int\limits_{2m_Q^2}^{2s_0} ds\, \exp\left(-\frac{s}{2M^2}\right) \times \left[ f^{(\mathrm{tw2})}\left(\frac{s}{m_Q^2} - 2\right) + \left(\frac{\mu_\pi}{m_Q}\right) f^{(\mathrm{tw3})}\left(\frac{s}{m_Q^2} - 2\right)\right], \qquad (4.13)$$

with the NLO contributions of twist-2 and twist-3:

$$\begin{aligned} f^{(\mathrm{tw2})}(\sigma) &= 3\left(3\,\ln\frac{m_Q^2}{\mu^2} - 4\right)\left[\delta(\sigma - 0^+) - \frac{1}{2}\right] + \frac{\pi^2}{2} \\ &+ 6\,\mathrm{Li}_2\left(-\frac{\sigma}{2}\right) - 3\,\mathrm{Li}_2\left(-\frac{\sigma}{\sigma+2}\right) + 3\,\mathrm{Li}_2\left(\frac{\sigma}{\sigma+2}\right) \\ &+ \ln\frac{\sigma}{2}\left[3\,\ln\frac{\sigma+2}{2} - \frac{3}{2}\,\frac{\sigma\,(\sigma+4)\,(3\,\sigma+10)+24}{(\sigma+2)^3}\right] \\ &+ 6\,\ln(\sigma+1)\frac{\sigma\,(\sigma+1)}{(\sigma+2)^3} - \frac{3}{4}\,\frac{3\,\sigma^2+20\,\sigma+20}{(\sigma+2)^2}\,, \end{aligned} \qquad (4.14)$$

$$\begin{aligned} f^{(\mathrm{tw3})}(\sigma) &= \left(3\,\ln\frac{m_Q^2}{\mu^2} - 4\right)\left[\delta(\sigma-0^+) + 2\,\delta'(\sigma-0^+)\right] + \left(\frac{4}{3}\,\pi^2 + 1\right)\delta(\sigma-0^+) + \frac{\pi^2}{3} \\ &+ 4\,\mathrm{Li}_2\left(-\frac{\sigma}{2}\right) - 2\,\mathrm{Li}_2\left(-\frac{\sigma}{\sigma+2}\right) + 2\,\mathrm{Li}_2\left(\frac{\sigma}{\sigma+2}\right) \\ &+ \ln\frac{\sigma}{2}\left[2\,\ln\frac{\sigma+2}{2} + \frac{\sigma^2+4}{2\,(\sigma+2)^2}\right] + 4\,\ln(\sigma+1)\frac{\sigma^2+2\,\sigma+2}{\sigma\,(\sigma+2)^2} \\ &+ \ln\frac{\sigma+2}{2}\left(\frac{\sigma}{8} - \frac{2}{\sigma}\right) + \frac{3\,\sigma^3+4\,\sigma^2-16\,\sigma-16}{16\,(\sigma+2)^2}\,, \end{aligned} \qquad (4.15)$$

where $\mathrm{Li}_2(x)$ is the Spence function. The expression for twist-2 part exactly matches the one given in [23], whereas the expression of the twist-3 NLO correction (4.15) obtained in the $\overline{\mathrm{MS}}$ scheme is a new result. To switch to the pole-mass scheme for the heavy quark, it is sufficient to add to (4.14) and (4.15) the terms $\Delta f^{(\mathrm{tw2})}(\sigma)$ and $\Delta f^{(\mathrm{tw3})}(\sigma)$, respectively, given in (B.11). As an additional check of our results, we have explicitly verified that the factorization-scale independence of both the twist-2 and twist-3 terms in the LCSR (4.5) at $O(\alpha_s^2)$ in the asymptotic limit.

The LCSR (4.5) for the strong $H^*H\pi$ coupling, where $H = B$ or $D$ and, respectively, $m_Q = m_b$ or $m_c$ with the LO and NLO terms given in (4.12) and (4.13) is now complete for the triangular duality region and ready for the numerical analysis.

## 5 Numerical results

To extract the strong couplings $g_{D^*D\pi}$ and $g_{B^*B\pi}$ from the LCSR (4.5), we need to divide out the decay constants of the pseudoscalar and vector heavy-light mesons. Here we will use two different procedures. The first one, applied in many LCSR applications, prescribes that, instead of adopting the fixed numerical values, one substitutes in (4.5) the two-point QCD sum rules for decay constants $f_H$ and $f_{H^*}$ $(H = D, B)$. These sum rules presented

JHEP03(2021)016

| parameter | input value | [Ref.] | rescaled values |
|---|---|---|---|
| quark-gluon coupling and quark masses | | | |
| $\alpha_s(m_Z)$ | $0.1179 \pm 0.0010$ | [3] | $\alpha_s(1.5\,\text{GeV}) = 0.3479^{+0.0100}_{-0.0096}$ <br> $\alpha_s(3.0\,\text{GeV}) = 0.2531^{+0.0050}_{-0.0048}$ |
| $\overline{m}_c(\overline{m}_c)$ | $1.280 \pm 0.025$ GeV | | $\overline{m}_c(1.5\,\text{GeV}) = 1.202 \pm 0.023$ GeV |
| $\overline{m}_b(\overline{m}_b)$ | $4.18 \pm 0.03$ GeV | | $\overline{m}_b(3.0\,\text{GeV}) = 4.46 \pm 0.04$ GeV |
| $(\overline{m}_u + \overline{m}_d)(2\ \text{GeV})$ | $6.78 \pm 0.08$ MeV | [3, 41] | $(\overline{m}_u + \overline{m}_d)(1.5\ \text{GeV}) = 7.40 \pm 0.09$ MeV <br> $(\overline{m}_u + \overline{m}_d)(3.0\ \text{GeV}) = 6.14 \pm 0.07$ MeV |
| condensates | | | |
| $\langle\bar{q}q\rangle(2\,\text{GeV})$ | $-\,(286 \pm 23\ \text{MeV})^3$ | [41] | $\langle\bar{q}q\rangle(1.5\,\text{GeV}) = -\,(279 \pm 22\ \text{MeV})^3$ <br> $\langle\bar{q}q\rangle(3.0\,\text{GeV}) = -\,(295 \pm 24\ \text{MeV})^3$ |
| $\langle GG\rangle$ | $0.012^{+0.006}_{-0.012}$ GeV$^4$ | [44] | — |
| $m_0^2$ | $0.8 \pm 0.2\,\text{GeV}^2$ | | — |
| $r_{vac}$ | $0.55 \pm 0.45$ | | — |

**Table 1**. QCD parameters used in the LCSRs and two-point sum rules.

in appendix C are taken from [28]. For consistency, following the arguments presented in [13, 23], the two-point sum rules are taken[2] at NLO, enabling a partial cancellation of perturbative corrections on both sides of (4.5). As a second, independent option, we will use the lattice QCD values for the charmed and bottom meson decay constants. Specifically, we will employ the latest $N_f = 2 + 1 + 1$ results: the averages for the heavy pseudoscalar mesons from [41] and the ratios of the vector and pseudoscalar meson decay constants obtained in [42],

$$f_D = 212.0 \pm 0.7\,\text{MeV}, \qquad f_B = 190.0 \pm 1.3\,\text{MeV},$$
$$f_{D^*}/f_D = 1.078 \pm 0.036, \qquad f_{B^*}/f_B = 0.958 \pm 0.022. \tag{5.1}$$

Furthermore, we have to specify the parameters entering the LCSR (4.5) and the auxiliary two-point sum rules for decay constants. The QCD input, including the quark-gluon coupling, the quark masses in $\overline{\text{MS}}$ scheme and the vacuum condensate densities, is listed in table 1. We adopt a very precise value of the light-quark mass combination $(m_u + m_d)$ determined in lattice QCD [41] (see the average in the quark-mass review of [3]). We also adopt the current interval of the quark condensate [41] which is consistent

[2]Note that in the previous analysis [23] the perturbative correction to the quark condensate contribution in the two-point sum rules was absent and is included now.

with the Gell-Mann-Oakes-Renner relation. The running of the QCD coupling and quark masses is performed with the four-loop accuracy [43] and the matching scales between $n_f = 5\,(n_f = 4)$ and $n_f = 4\,(n_f = 3)$ are, respectively 4.2 GeV and 1.3 GeV.

Let us discuss now our choice for the input parameters of pion DAs. In the LO part (4.12) of the LCSR, we encounter the values of the DAs or their derivatives at the middle point $u = 1/2$. Note that the midpoint value of a given DA is determined by a complete set of the coefficients in the conformal expansion, so that the LCSR (4.5) provides an additional source of information on the structure of DAs. In this respect it is different from the LCSRs for the $B \to \pi$ and $D \to \pi$ form factors, where the pion DAs are weighted by the Borel exponent and integrated over the duality interval. On the other hand, since the NLO part of the LCSR is calculated for the asymptotic twist-2 and twist-3 two-particle DAs, the only inputs necessary for a numerical evaluation of (4.13) are the normalization factors of these DAs given, respectively, by the pion decay constant $f_\pi$ and by the parameter $\mu_\pi$ defined in (2.5).

The key parameter of the LO twist-2 part of LCSR (4.5) is the value of $\varphi_\pi(1/2, \mu)$. Expanding this DA in the Gegenbauer polynomials according to (A.1), we find

$$\varphi_\pi(1/2,\, 1\,\text{GeV}) = 1.5 - 2.25\, a_2 + 2.8125\, a_4 - 3.28125\, a_6 + 3.69141\, a_8 + \dots\,. \tag{5.2}$$

Hereafter, unless the renormalization scale $\mu$ is explicitly shown, we denote by $a_n$ the Gegenbauer moments taken at the scale $\mu = 1\,\text{GeV}$. We see that the midpoint value of the twist-2 DA contains a sign alternating series of all Gegenbauer moments with slowly growing numerical coefficients. At larger scales, the moments decrease, e.g.:

$$\varphi_\pi(1/2,\, 3\,\text{GeV}) \simeq 1.5 - 1.471\, a_2 + 1.515\, a_4 - 1.553\, a_6 + 1.585\, a_8 + \dots\,, \tag{5.3}$$

where the scale dependence calculated using (A.2) is absorbed in the numerical coefficients. At $\mu \to \infty$, the value of $\varphi_\pi(1/2)$ approaches its asymptotic limit equal to $3/2$. Still, at finite scales, $\varphi_\pi(1/2)$ is an important indicator of the nonasymptotic effects, complementing the available knowledge of the lowest Gegenbauer moments.

Currently, only the second moment $a_2$ of the pion DA is accessible in QCD on the lattice. We will use the latest quite accurate result:

$$a_2(2\,\text{GeV}) = 0.116^{+0.019}_{-0.020} \tag{5.4}$$

obtained in [45]. From the same analysis, higher Gegenbauer moments cannot be extracted reliably, e.g. for $a_4$ only a preliminary value is quoted, which will not be considered here. To estimate and/or constrain the values of $a_{n\geq 4}$, one has to resort to the phenomenologically viable models of $\varphi_\pi(u)$ expanding them in Gegenbauer polynomials.

To choose the input value of $\varphi_\pi(1/2)$, we adopt two such models. The first one denoted here as Model 1 was suggested in [45]:

$$\text{Model 1}: \qquad \varphi_\pi(u) = \frac{\Gamma(2+2\alpha_\pi)}{[\Gamma(1+\alpha_\pi)]^2}\, u^{\alpha_\pi}\,(1-u)^{\alpha_\pi}\,. \tag{5.5}$$

| Twist | Parameter | input value | Source [Ref.] | rescaled values |
|---|---|---|---|---|
| 2 | $f_\pi$ | $130.4\,\mathrm{MeV}$ | [3] | — |
| | $\varphi_\pi(1/2, 2\,\mathrm{GeV})$ | $1.31 \pm 0.03$ | Model 1 [45] | $\varphi_\pi(1/2, 1.5\,\mathrm{GeV}) = 1.31^{+0.03}_{-0.02}$<br>$\varphi_\pi(1/2, 3.0\,\mathrm{GeV}) = 1.34^{+0.02}_{-0.02}$ |
| | $\varphi_\pi(1/2, 1\,\mathrm{GeV})$ | $0.99 \pm 0.36$ | Model 2 [51] | $\varphi_\pi(1/2, 1.5\,\mathrm{GeV}) = 1.09 \pm 0.26$<br>$\varphi_\pi(1/2, 3.0\,\mathrm{GeV}) = 1.18 \pm 0.19$ |
| 3 | $\mu_\pi(2\,\mathrm{GeV})$ | $2.87 \pm 0.03$ GeV | $\frac{m_\pi^2}{m_u+m_d}$ [3, 41] | $\mu_\pi(1.5\,\mathrm{GeV}) = 2.63 \pm 0.03$ GeV<br>$\mu_\pi(3.0\,\mathrm{GeV}) = 3.17 \pm 0.04$ GeV |
| | $f_{3\pi}(1\,\mathrm{GeV})$ | $(4.5 \pm 1.5) \cdot 10^{-3}\,\mathrm{GeV}^2$ | [34] | $f_{3\pi}(1.5\,\mathrm{GeV}) = (3.6 \pm 1.2) \cdot 10^{-3}\,\mathrm{GeV}^2$<br>$f_{3\pi}(3.0\,\mathrm{GeV}) = (2.8 \pm 0.9) \cdot 10^{-3}\,\mathrm{GeV}^2$ |
| | $\omega_{3\pi}(1\,\mathrm{GeV})$ | $-1.5 \pm 0.7$ | | $\omega_{3\pi}(1.5\,\mathrm{GeV}) = -1.2 \pm 0.6$<br>$\omega_{3\pi}(3.0\,\mathrm{GeV}) = -1.0 \pm 0.5$ |
| 4 | $\delta_\pi^2(1\,\mathrm{GeV})$ | $0.18 \pm 0.06\,\mathrm{GeV}^2$ | [34] | $\delta_\pi^2(1.5\,\mathrm{GeV}) = 0.16 \pm 0.05$<br>$\delta_\pi^2(3.0\,\mathrm{GeV}) = 0.14 \pm 0.05$ |
| | $\epsilon_\pi(1\,\mathrm{GeV})$ | $0.5 \pm 0.3$ | | $\epsilon_\pi(1.5\,\mathrm{GeV}) = 0.4 \pm 0.2$<br>$\epsilon_\pi(3.0\,\mathrm{GeV}) = 0.3 \pm 0.2$ |

**Table 2**. Parameters of pion DAs.

Its single free parameter is fixed by equating the second Gegenbauer moment of this model to the lattice QCD result (5.4), yielding $\alpha_\pi(2\,\mathrm{GeV}) = 0.585^{+0.061}_{-0.055}$. In addition, the first inverse moment of this DA is

$$\int_0^1 du\, \frac{\varphi_\pi(u, 2\,\mathrm{GeV})}{1-u} = 2 + \frac{1}{\alpha_\pi(2\,\mathrm{GeV})} = 3.71^{+0.18}_{-0.16}\,. \tag{5.6}$$

The corresponding midpoint value of the DA (5.5) is given in table 2. Note that the inverse moment serves as the main input in the QCD calculation of the photon-pion transition form factor [46–49]. As noted in [45], applying this method with the above value, one achieves a good description of data on this form factor.

Our Model 2 is of a different origin and is based on the comparison of the LCSR for the pion electromagnetic form factor [50] with the experimental data. We use the results of the recent analysis [51], where a dispersion relation and the data in the timelike region are used to reproduce the pion form factor in the spacelike region. These results are then used to fit the LCSR form factor calculated to the twist-2 NLO accuracy including the subleading twist-4,6 terms. Among various versions of the fitted twist-2 DAs we choose the optimal one with the first three moments in the Gegenbauer expansion (A.1). The fit results obtained in [51] at the scale of 1 GeV are

$$\text{Model 2}: \quad a_2 = 0.270 \pm 0.047, \quad a_4 = 0.179 \pm 0.060, \quad a_6 = 0.123 \pm 0.086\,, \tag{5.7}$$

with the correlation matrix:

$$\begin{pmatrix} 1.0 & -0.15 & -0.13 \\ -0.15 & 1.0 & -0.13 \\ -0.13 & -0.13 & 1.0 \end{pmatrix}. \tag{5.8}$$

The corresponding input value of $\varphi_\pi(1/2, 1\,\text{GeV})$ is given in table 2.

In the same table we specify the input parameters entering the pion twist-3 and twist-4 DAs presented in appendix A. These DAs were worked out in [34] to the next-to-leading order of conformal expansion. Their normalization and nonasymptotic coefficients at $\mu = 1\,\text{GeV}$ used as an input here were calculated from the two-point QCD sum rules (see [34] and references therein). We notice, in particular, the relative smallness of the twist-3 parameter $f_{3\pi}$, which determines the nonasymptotic part of the two-particle DAs and the normalization of the three-particle DA. Hence, the large twist-3 contribution to LCSR is, to a good precision, determined by the asymptotic two-particle DAs $\phi^p_{3\pi}$ and $\phi^\sigma_{3\pi}$ at the midpoint. Here we greatly benefit from the very accurate value of the twist-3 normalization parameter $\mu_\pi$ which is determined by the light-quark masses. Note at the same time that the $O((m_u + m_d)^2/m_\pi^2)$ correction to the ratio of normalization factors for $\phi^\sigma_{3\pi}$ and $\phi^p_{3\pi}$ is still small enough to be neglected safely. The contributions of the twist-4 two- and three-particle DAs, as we will see, are altogether strongly suppressed. Therefore, there is no compelling reason to go beyond the current accuracy, and e.g., calculate the NLO corrections to the twist-4 part, which is technically a challenging task.

To complete the choice of the input, we take the meson masses from [3], considering, for definiteness, the strong coupling $\langle D^{*+}\pi^-|D^0\rangle$ and, correspondingly, $\langle \bar{B}^{*0}\pi^-|B^-\rangle$ in (2.1). All other couplings with different combinations of charges are related to the above ones via the isospin symmetry (see e.g. [13]). Finally, we specify the variable parameters of the LCSR (4.5), which include: the renormalization scale of the quark-gluon coupling and quark masses, the factorization scale, the Borel parameters and the quark-hadron duality thresholds. Since we perform the calculations at finite masses, these scales and thresholds are evidently different in the sum rules involving charmed and bottom mesons. On the other hand, heavy-quark spin symmetry allows us to equate certain scales, most importantly, the Borel parameters in the $H$ and $H^*$ channels. The chosen default values and intervals of all relevant scales and thresholds are presented in table 3. Here we follow the numerical analysis of the related LCSRs for the $D \to \pi$ and $B \to \pi$ form factors. More specifically, we employ for the charm and bottom cases of (4.5) the same variable scales and thresholds as, respectively, in [22] and [52]. The compelling argument is that we deal here with the same underlying correlation function and the same light-cone OPE as in the form factor sum rules. Also, the renormalization scales of $\alpha_s$ and quark masses are taken equal to the factorization scale $\mu$ appearing in the OPE of the correlation function[3] (2.3).

[3]Note that the currents $j_\mu$ and $j_5$ in (2.3) are renormalization invariant.

JHEP03(2021)016

| Parameter | default value (interval) | [Ref.] |
|---|---|---|
| charmed meson sum rules | | |
| $\mu$ (GeV) | 1.5 (1.0 - 3.0) | |
| $M^2$ (GeV$^2$) | 4.5 (3.5 - 5.5) | [22] |
| $s_0$ (GeV$^2$) | 7.0 (6.5 - 7.5) | |
| $\bar{M}^2$(GeV$^2$) | 2.0 (1.5 - 2.5) | |
| $\bar{s}_0$ (GeV$^2$) | 5.6 | [28] |
| $\bar{s}_0^*$(GeV$^2$) | 6.2 | |
| bottom meson sum rules | | |
| $\mu$ (GeV) | 3.0 (2.5 - 4.5) | |
| $M^2$ (GeV$^2$) | 16.0 (12.0 - 20.0) | [52] |
| $s_0$ (GeV$^2$) | 37.5 (35.0 - 40.0) | |
| $\bar{M}^2$ (GeV$^2$) | 5.5 (4.5 - 6.5) | |
| $\bar{s}_0$(GeV$^2$) | 33.9 | [28] |
| $\bar{s}_0^*$(GeV$^2$) | 34.1 | |

**Table 3**. The renormalization scale $\mu$, Borel parameters $M^2$ and $\bar{M}^2$ and duality thresholds $s_0$ and $\bar{s}_0$ ($\bar{s_0}^*$) used, respectively in the LCSR and the two-point sum rules for the $H$ ($H^*$) decay constants for both charmed and bottom mesons.

In the adopted approximation the factorization scale reveals itself in the nonasymptotic components of DAs in the LO part, while in the NLO part we use the asymptotic DAs. For consistency, the same scale is used in the corresponding two-point sum rules for $f_H$ and $f_{H^*}$.

In tables 1 and 2, apart from the input values of the scale-dependent parameters at a given reference scale $\mu = 2.0\,$GeV or $\mu = 1.0\,$GeV, we also present, for convenience, their rescaled values at $\mu = 1.5\,$GeV and $\mu = 3.0\,$GeV, to be used in the sum rules with charmed and bottom mesons, respectively. Note that the midpoint value of the twist-2 DA can be rescaled only if it is expressed as a linear combination of multiplicatively renormalizable Gegenbauer moments, because the latter possess different anomalous dimensions. Hence, for the Model 1 we first calculate the Gegenbauer moments of the DA in (5.5), and then, forming the expansion, rescale each moment according to (A.2). For the Model 2 the rescaling is straightforward. The resulting values of $\varphi_\pi(1/2)$ for both models are presented in table 2 at the two default scales, together with the parameters used for the twist-3 and twist-4 DAs. The formulas determining the scale dependence of the latter can be found e.g. in the appendix A of [21]. As already mentioned above, the intervals of Borel parameter, as well as the values of threshold parameters in the LCSR (4.5) are the same as in the LCSR analyses for $D \to \pi$ [22] and $B \to \pi$ [52] form factors. In each of these analyses, the duality threshold was adjusted by fitting the differentiated sum rule to the heavy meson mass. The Borel parameters and duality thresholds in the two-point sum rules for the $H^{(*)}$ decay constants are taken from [28].

JHEP03(2021)016

| LCSR result | tw 2 LO | tw 2 NLO | tw 3 LO | tw 3 NLO | tw 4 | total |
|---|---|---|---|---|---|---|
| $f_D\, f_{D^*}\, g_{D^*D\pi}$ [GeV$^2$] | 0.188 (Model 1)<br>0.156 (Model 2) | 0.049 | 0.333 | 0.115 | -0.001 | 0.684<br>0.652 |
| $f_B\, f_{B^*}\, g_{B^*B\pi}$ [GeV$^2$] | 0.416 (Model 1)<br>0.367 (Model 2) | 0.081 | 0.395 | 0.148 | -0.004 | 1.037<br>0.988 |

**Table 4**. Numerical results at the central input.

| LCSR result | $\alpha$ | tw 2 NLO | tw 3 NLO |
|---|---|---|---|
| $f_D\, f_{D^*}\, g_{D^*D\pi}$ [GeV$^2$] | 1/2 | 0.056 | 0.128 |
| | 1 | 0.049 | 0.115 |
| | 2 | 0.038 | 0.093 |
| $f_B\, f_{B^*}\, g_{B^*B\pi}$ [GeV$^2$] | 1/2 | 0.090 | 0.158 |
| | 1 | 0.081 | 0.148 |
| | 2 | 0.066 | 0.131 |

**Table 5**. Numerical results for different duality regions at central values of parameters.

With the input specified above, we calculate first the product of the strong coupling and decay constants from the sum rule (4.5). The results are presented in table 4 at the central input and at default scales, including also the separate twist and NLO contributions. The twist-2 and twist-3 contributions are at the same level, similar as in the LCSRs for heavy-to-light form factors. In the twist-2 LO part of LCSR (4.5) the contributions of nonasymptotic terms are quite noticeable, as can be seen from comparison of the results for the two different DA models. At the same time, the share of asymptotic DAs constitutes 93% (87%) of the twist-3 LO contribution for the bottom (charmed) meson case. The convergence of the OPE is supported by the smallness of the twist-4 contributions. In addition, as already mentioned, the twist-5 and twist-6 terms in the OPE of the correlation function (2.3) obtained in the factorizable approximation in [26] have negligible impact, allowing us to neglect them here. On the other hand, as seen from table 4, the NLO contributions are appreciable, reaching e.g. for the bottom meson case the level of 20% (35%) for twist 2 (twist 3). The results presented in this table correspond to our default choice of the triangle duality region in the $(s_1, s_2)$ plane, described by the parameterization (4.2) at $\alpha = 1/2$. In addition, to investigate how the choice of the duality region influences the LCSR, we calculated the NLO terms at the same default value of $s_0$ for two other choices of the region corresponding to $\alpha = 1$ and $\alpha = 2$. The results are presented in table 5. We see that deviations with respect to the choice of triangle region are at the level of a few percent of the total values of both $f_D f_{D^*} g_{D^*D\pi}$ and $f_B f_{B^*} g_{B^*B\pi}$. We include this deviation into the total uncertainty for the predicted strong couplings $g_{D^*D\pi}$ and $g_{B*B\pi}$.

To finally obtain these couplings, we divide out the heavy-meson decay constants applying the two different methods described above: we either use the two-point sum rules or the lattice QCD results listed in (5.1). For comparison, we quote the values of decay

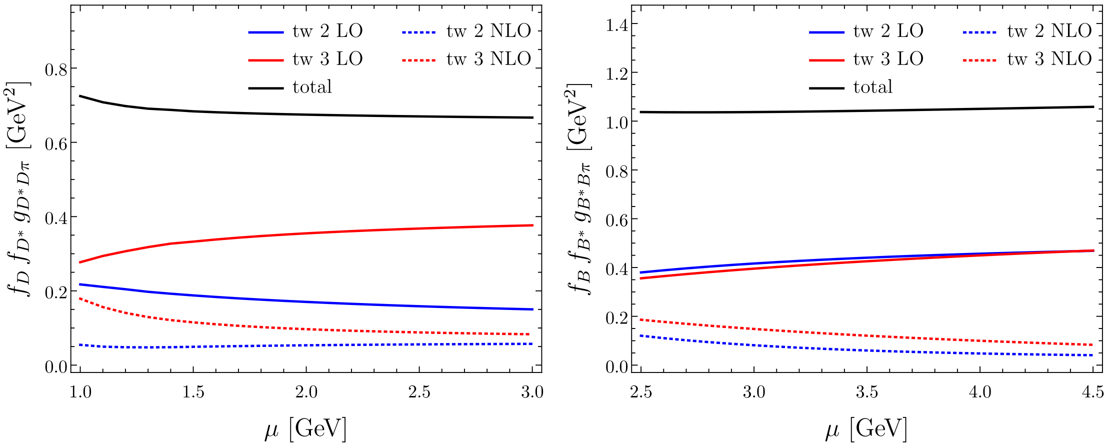

**Figure 2**. Scale dependence of the products $f_D f_{D^*} g_{D^*D\pi}$ and $f_B f_{B^*} g_{B^*D\pi}$ calculated from LCSR. Displayed are the total values and separate twist-2 and twist-3 contributions for Model 1 of the twist-2 pion DA and at central values of all other input parameters.

constants calculated at NLO from the two-point sum rules at central input:

$$f_D = 190.7 \text{ MeV}, \qquad f_{D^*} = 247.3 \text{ MeV}, \qquad f_B = 201.0 \text{ MeV}, \qquad f_{B^*} = 214.0 \text{ MeV}\,.$$

Our final results are shown in table 6 for both Model 1 and 2 of the twist-2 DA and for both choices of the decay constants. The interval attributed to each separate entry in table 6 is evaluated, adding in quadrature the separate uncertainties caused by individual variations of all input parameters and scales within their adopted intervals in LCSR. When using the two-point sum rules for the $D^{(*)}$ and $B^{(*)}$ decay constants, we vary the scale $\mu$ and Borel parameters (condensate densities) concertedly (independently). The lattice results for the decay constants have very small errors which play almost no role in the uncertainty budget. Finally, the total uncertainty quoted in table 6 also includes the variation due to the change of the duality region in the NLO part as described in section 4. We assume that the latter uncertainty at least partially assesses the “systematic error” of LCSR caused by the quark-hadron duality ansatz.

The LCSR prediction for $D^*D\pi$ ($B^*B\pi$) strong coupling has altogether an estimated uncertainty of 20-25% (15-20%), if the heavy-meson decay constants are replaced by the two-point sum rules. The uncertainties become smaller when we use more accurate lattice QCD values for the heavy-meson decay constants. For the two options for decay constants the predicted intervals of $g_{D^*D\pi}$ are close to each other, whereas the intervals of $g_{B^*B\pi}$ only marginally agree. The shift between the central values of the $B^*B\pi$ coupling mainly originates due to the $\mathcal{O}\,(20\%)$ difference between the lattice-QCD value of $f_{B^*}$ and the central value of the two-point sum rule prediction.[4] We also notice that the choice of the

[4] As already mentioned, for consistency, here we use the two-point sum rules at NLO. More accurate sum rules at NNLO yield [28] the ratio $f_{B^*}/f_B = 1.02^{+0.02}_{-0.09}$, reflecting the heavy-spin symmetry breaking effect. Within uncertainties, this result is in agreement with (5.1) calculated in the lattice QCD [42].

JHEP03(2021)016

| $\varphi_\pi(1/2)$ | decay constants | $g_{D^*D\pi}$ | $g_{B^*B\pi}$ | $\hat{g}$ | $\delta$ [GeV] |
|---|---|---|---|---|---|
| Model 1 | 2-point sum rule | $14.5^{+3.5}_{-2.4}$ | $24.1^{+4.5}_{-3.8}$ | $0.18^{+0.02}_{-0.03}$ | $3.28^{+0.62}_{-0.17}$ |
| | Lattice QCD | $14.1^{+1.3}_{-1.2}$ | $30.0^{+2.6}_{-2.4}$ | $0.30^{+0.02}_{-0.02}$ | $1.17^{+0.04}_{-0.04}$ |
| Model 2 | 2-point sum rule | $13.8^{+3.1}_{-2.3}$ | $23.0^{+4.5}_{-3.8}$ | $0.17^{+0.03}_{-0.03}$ | $3.31^{+0.30}_{-0.01}$ |
| | Lattice QCD | $13.5^{+1.4}_{-1.4}$ | $28.6^{+3.0}_{-2.8}$ | $0.29^{+0.03}_{-0.03}$ | $1.18^{+0.00}_{-0.02}$ |

**Table 6**. LCSR results for the strong couplings of the charmed and bottom mesons for the two methods of dividing out the decay constants and the two models of the pion twist-2 DA, at the central values of parameters.

model for the pion twist-2 DA is less important for the charmed-meson strong coupling, the reason being a dominance of the twist-3 contribution enhanced by the ratio $\mu_\pi/m_c$ with respect to $\mu_\pi/m_b$ for the bottom-meson coupling.

Comparing our numerical results with the original LCSR calculation in [13], we notice a substantial increase of the products of the strong couplings and decay constants displayed in table 4 with respect to the results $f_D f_{D^*} g_{D^*D\pi} = 0.51 \pm 0.05$ GeV$^2$ and $f_B f_{B^*} g_{B^*B\pi} = 0.64 \pm 0.06$ GeV$^2$ obtained in [13]. This increase is mainly caused by the twist-2 and twist-3 NLO terms in the LCSR, which were absent in [13]. In addition, the updated input parameters in our numerical analysis differ from the ones adopted in [13]. Most importantly, we use the $\overline{\text{MS}}$ heavy-quark masses instead of the pole-mass scheme employed in [13]. In particular, the updated value of the twist-3 normalization parameter $\mu_\pi$ has become substantially larger. Moreover, the updated inputs affect the numerical LO result in different directions, largely compensating each other in the case of charmed mesons and generating an additional increase in the case of bottom mesons. The determined strong couplings are numerically influenced by the magnitude of the products of heavy-meson decay constants. In [13] they were taken from the two-point sum rules at LO. By contrast, the larger values of these products are employed here such that the above-mentioned increase of the LCSR results is either partially (for the charmed-meson case) or almost completely (for the bottom-meson case) compensated, as can be seen by comparing our predictions presented in table 6 with $g_{D^*D\pi} = 12.5 \pm 1.0$ and $g_{B^*B\pi} = 29 \pm 3$ from [13].

It is also instructive to investigate the limit of infinitely heavy-quark mass obtained from the LCSR (4.5) for the strong coupling. This sum rule, where the underlying correlation function is calculated at the finite mass $m_Q$, not only reproduces the leading-power behaviour of the coupling at $m_Q \to \infty$ but also enables a quantitative assessment of the $1/m_Q$ corrections. For the heavy-to-light form factors obtained from the LCSRs, the heavy-quark mass expansion has been investigated in the early papers [53, 54]. To proceed, we apply to the sum rule (4.5) the usual scaling relations (valid up to the inverse heavy-quark mass corrections):

$$f_H = f_{H*} = \frac{\hat{f}}{\sqrt{m_Q}}, \quad m_H = m_{H*} = m_Q + \overline{\Lambda}, \quad M^2 = 2m_Q\tau, \quad s_0 = m_Q^2 + 2m_Q\omega_0\,, \tag{5.9}$$

JHEP03(2021)016

where $\hat{f}$ and $\overline{\Lambda}$ are, respectively, the static decay constant and the binding energy of heavy meson in HQET, and the parameters $\tau$ and $\omega_0$ do not scale with $m_Q$. We obtain at LO

$$g_{H^*H\pi} = \frac{2m_Q}{f_\pi}\left[\frac{f_\pi^2}{\hat{f}^2}e^{\overline{\Lambda}/\tau}\left\{\tau\left(1-e^{-\omega_0/\tau}\right)\varphi_\pi(1/2)+\frac{\mu_\pi}{6}\phi_{3\pi}^\sigma(1/2)-\frac{\phi_{4\pi}(1/2)}{16\tau}\right\}\right]+\dots\,, \quad (5.10)$$

where ellipsis indicates the inverse heavy-mass corrections. Comparing the above formula with the definition (2.2) and taking the limit $m_Q \to \infty$, we notice that the expression in square brackets is nothing but a sum rule for the static coupling $\hat{g}$ in HM$\chi$PT. Note that the twist-3 and 4 terms also contribute to this sum rule. Moreover, from the LCSR (4.5) we are in a position to estimate both the static coupling and the inverse mass corrections to it. Including the NLO terms in this limiting procedure is however nontrivial, because one has to resum the logarithms of the heavy-quark mass. A systematic way is to derive the LCSR for the strong coupling directly in HQET, a task which is beyond our scope here.

Expanding the rescaled sum rule (4.5) in the powers of $1/m_Q$, we follow [13], and parameterize the LCSR result for the strong coupling a form (2.2) with an added inverse heavy-mass correction:[5]

$$g_{H^*H\pi} = \frac{2m_H\hat{g}}{f_\pi}\left(1+\frac{\delta}{m_H}\right), \quad (H=D,B)\,. \quad (5.11)$$

Equating the above formula to the obtained values of $g_{D^*D\pi}$ and $g_{B^*B\pi}$ presented in table 6, we encounter the two equations yielding the parameters $\hat{g}$ and $\delta$. Their resulting values are presented in the last two columns of the same table. We find that the inverse mass corrections are large, especially in the case of the $D^*D\pi$ strong coupling as it was already noticed in [13]. Hence, estimating the static coupling $\hat{g}$ from the known $D^*D\pi$ or $B^*B\pi$ couplings via the relation (2.2), as it is frequently done in the literature, is actually not reliable in practice.

The obtained values for the $D^*D\pi$ coupling can be compared to its experimentally measured value, extracted from the width of the $D^{*+}\to D^0\pi^+$ decay:

$$\Gamma(D^{*+}\to D^0\pi^+) = \frac{g^2_{D^*D\pi}}{24\pi m^2_{D^*}}|\vec{p}|^3\,, \quad (5.12)$$

where $|\vec{p}| = 39\,\text{MeV}$ is the decay momentum in the rest frame of $D^*$. The above formula for the partial width is obtained from the decay amplitude which is defined by crossing-transforming the initial definition (2.1)

$$\langle D^0(q-p)\pi^+(p)|D^{*+}(q)\rangle = -g_{D^*D\pi}\,p^\mu\epsilon_\mu^{(D^*)}\,. \quad (5.13)$$

The PDG average [3] of the two measurements [1, 2] of the $D^{*\pm}$-meson total width is $\Gamma_{\text{tot}}(D^{*\pm}) = 83.4\pm1.8\,\text{keV}$[6] and using the precisely measured branching fraction $\mathcal{BR}(D^*\to$

[5]This parametrization is further supported by the heavy quark expansion of the strong couplings $H^*H\pi$ in the framework of HM$\chi$PT [55, 56].

[6]For the $D^{*0}$ total width only an upper limit is measured so far. To relate the total widths of charged and neutral $D^*$ mesons, the isospin symmetry is not sufficient, because one needs in addition the radiative decay widths $\Gamma(D^*\to D\gamma)$. Currently, the latter are only available from the theory estimates. The LCSR prediction can be found e.g. in [36, 57].

JHEP03(2021)016

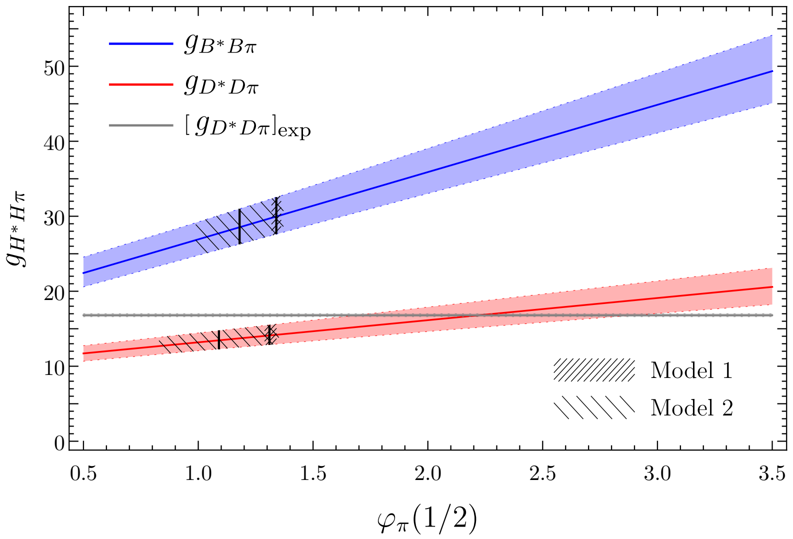

**Figure 3**. The dependence of $g_{D^*D\pi}$ and $g_{B^*B\pi}$ on the value of $\varphi_\pi(1/2)$. The lattice QCD results for the decay constants of heavy mesons are employed.

$D\pi) = 0.677 \pm 0.005$ from [3] yields

$$\Gamma(D^{*+} \to D^0\pi^+) = (56.5 \pm 1.3)\,\text{keV}\,, \tag{5.14}$$

where we have added the two errors of independent measurements in quadrature. Then, from (5.13) we finally obtain the strong coupling:

$$[g_{D^*D\pi}]_{\rm exp} = 16.8 \pm 0.2\,. \tag{5.15}$$

Our results on the $D^*D\pi$ coupling presented in table 6 are somewhat smaller than the above value, but the difference is not significant. Even if we take the smallest interval predicted from LCSR (the combination of Model 2 with the lattice decay constants) its upper limit is only 10% smaller than the measured strong coupling.

Furthermore, it is instructive to investigate how sensitive are the LCSRs for $D^*D\pi$ and $B^*B\pi$ couplings to the midpoint value of the pion twist-2 DA. In figure 3 we plot the dependence of both strong couplings on $\varphi_\pi(1/2)$ considering the latter as a free parameter. We observe a very mild dependence of $g_{D^*D\pi}$, so that the overlap of the LCSR prediction within its uncertainty interval with the experimental value (5.15) yields a broad interval with $\varphi_\pi(1/2) > 1.5$. Having in mind that an unaccounted uncertainty of LCSR at the level of $\sim 10\%$ is not excluded, we conclude that fixing the midpoint value of $\varphi_\pi$ only from the measured $D^*D\pi$ coupling is not realistic. In this respect, the dependence of $g_{B^*B\pi}$ on $\varphi_\pi(1/2)$ plotted in figure 3 is steeper. Hence, an accurate lattice QCD prediction for this coupling obtained at finite $b$-quark mass, being equated to LCSR, can yield a more tight constraint on the pion DA.

Returning to the comparison of $g_{D^*D\pi}$ with experiment, a comment is in order. The interval $\varphi_\pi(1/2, \mu = 1.5\,\text{GeV}) > 1.5$ preferred from this comparison may indicate that the

JHEP03(2021)016

| Method | $g_{D^*D\pi}$ | $g_{B^*B\pi}$ | $\hat{g}$ |
|---|---|---|---|
| LQCD, $N_f = 2$ [8] | $15.9 \pm 0.7^{+0.2}_{-0.4}$ | — | — |
| LQCD, $N_f = 2+1$ [9] | $16.23 \pm 1.71$ | — | — |
| LQCD, $N_f = 2+1$ [12] | — | $\frac{2m_B}{f_\pi}(0.56 \pm 0.03 \pm 0.07)$ $= 45.3 \pm 6.0$ | — |
| LQCD, $N_f = 2$ [7] | — | — | $0.44 \pm 0.03^{+0.07}_{-0.0}$ |
| LQCD, $N_f = 2+1$ [10] | — | — | $0.449 \pm 0.051$ |
| LQCD, $N_f = 2$ [11] | — | — | $0.492 \pm 0.029$ |
| LCSR (this work) | $14.1^{+1.3}_{-1.2}$ | $30.0^{+2.6}_{-2.4}$ | $0.30^{+0.02}_{-0.02}$ |

**Table 7**. Strong couplings of the heavy mesons with pion obtained from lattice QCD (LQCD), compared with our LCSR prediction (using Model 1 of the pion DA and the lattice-QCD decay constants).

pion twist-2 DA at low scales has a structure different from both Models 1, 2 we have used. It is in fact possible to construct a pion DA which has a midpoint value exceeding the asymptotic limit and, simultaneously, the second Gegenbauer moment equal to the lattice QCD value in (5.4). The simplest option is to adopt a truncated Gegenbauer expansion with a relatively large positive $a_4(1\,\text{GeV})$ and small $a_{n>4}$. A pion DA with such a pattern of Gegenbauer moments at $\mu = 1\,\text{GeV}$: $a_2 = 0.135 \pm 0.032$ (the rescaled value (5.4)) and $a_4 = 0.218 \pm 0.059$, $a_{n>4} = 0$, was used in [51] (see the second line in the table IV there) among other models fitting the LCSR for the pion electromagnetic form factor to the timelike form factor data. According to (5.2), the resulting midpoint value is $\varphi_\pi(1/2, \mu = 1\,\text{GeV}) = 1.81 \pm 0.18$. Note however that the Model 2 chosen above and adopted from the same analysis provides a better fit to the pion form factor.

In table 7 we compare our results with the strong couplings calculated from the lattice QCD. We only select the results obtained with the number of flavours $N_f > 1$. The determinations of $g_{D^*D\pi}$ in [8, 9] are consistent with our results, whereas the only available result [12] for $g_{B^*B\pi}$ is significantly larger than our prediction. The same is valid for the static coupling $\hat{g}$ in the lattice QCD. The latter comparison is, however, not completely consistent because our results for $\hat{g}$ are based on the fit of eq. (5.11) which includes the higher-order correction in the heavy quark expansion.

Furthermore, let us mention an independent possibility to extract the $B^*B\pi$ coupling. The procedure, explained in detail in [58] (see also [59]), is based on the hadronic dispersion

JHEP03(2021)016

relation for the $B \to \pi$ vector form factor:

$$f^+_{B\pi}(q^2) = \frac{g_{B^*B\pi} f_{B^*}}{2m_{B^*}(1-q^2/m^2_{B^*})} + \frac{1}{\pi} \int\limits_{(m_B+m_\pi)^2}^{\infty} dt \, \frac{\text{Im} f^+_{B\pi}(t)}{t-q^2} \tag{5.16}$$

valid without subtractions. The above relation contains the vector-meson $B^*$ pole, which lies slightly below the threshold[7] $q^2 = (m_B+m_\pi)^2$. A small width of this meson determined by the $B^* \to B\gamma$ decay can safely be neglected in (5.16). The residue of the $B^*$ pole is a product of the $B^*B\pi$ coupling and the $B^*$ decay constant. Multiplying both sides of (5.16) by the denominator of the pole term, we take the limit $q^2 \to m^2_{B^*}$, removing the complicated integral over hadronic spectral density, so that

$$g_{B^*B\pi} = \frac{2m_{B^*}}{f_{B^*}} \lim_{q^2 \to m^2_{B^*}} \left[ \left(1 - q^2/m^2_{B^*}\right) f^+_{B\pi}(q^2) \right]. \tag{5.17}$$

Here we benefit from the fact that the pion mass is much smaller than $m_B$, hence, any analytic representation of the expression in the square bracket of the above equation, valid in the low pion recoil region $q^2 \lesssim (m_B - m_\pi)^2$ of the $B \to \pi$ transition, provides an accurate limit. The most convenient representation for that purpose is the BCL version [60] of the $z$-expansion based on the conformal mapping of the momentum transfer squared:

$$q^2 \to z(q^2) = \frac{\sqrt{(m_B+m_\pi)^2 - q^2} - \sqrt{(m_B+m_\pi)^2 - t_0}}{\sqrt{(m_B+m_\pi)^2 - q^2} + \sqrt{(m_B+m_\pi)^2 - t_0}} \,,$$

where the optimal choice is $t_0 = (m_B+m_\pi)(\sqrt{m_B} - \sqrt{m_\pi})^2$. In this case, the expression under the square bracket in (5.17) represents a polynomial in $z$ and can be easily continued to $z(m^2_{B^*})$.

As an example, we make use of the lattice QCD, $N_f = 2+1$ calculation [61] of the $B \to \pi$ form factor, where the $z$-expansion

$$\left(1 - q^2/m^2_{B^*}\right) f^+_{B\pi}(q^2) = \sum_{n=0}^{N_z-1} b^+_n \left( [z(q^2)]^n - (-1)^{n-N_z} \frac{n}{N_z} [z(q^2)]^{N_z} \right), \tag{5.18}$$

was implemented. Choosing the preferred $N_z = 4$ version with the coefficients $b^+_n$ from table XIV of [61], we substitute (5.18) in (5.17). Adopting for $f_{B^*}$ the lattice QCD value in (5.1), we obtain:

$$g_{B^*B\pi} = 34.5 \pm 3.0 \,, \tag{5.19}$$

where the uncertainty is obtained taking into account the errors and correlations of the coefficients $b^+_n$ quoted in [61] and the error of the decay constant $f_{B^*}$.

Before commenting on this result, we note that the truncated $z$-expansion in (5.18) is employed here merely as a smooth fit function. It is used to fit the l.h.s. of (5.18) which consists of the form factor calculated [61] at $17\,\text{GeV}^2 < q^2 < 26\,\text{GeV}^2$ and multiplied with $(1 - q^2/m^2_{B^*})$ to remove the $B^*$ pole . We then extrapolate this function to a slightly larger

[7] The separation of the $D^*$ pole located above the $D\pi$ threshold is not a straightforward procedure, hence here we refrain from considering the same method for the $D^*D\pi$ coupling.

JHEP03(2021)016

$q^2 = m_{B^*}^2 \simeq 28\,\text{GeV}^2$ to reach the limit (5.17). Hence, the accuracy of the truncation in (5.18) plays a minor role in this procedure, being more important for an extrapolation to the small $q^2$ region.

The estimate (5.19) turns out to be significantly smaller than the result of a "direct" lattice QCD calculation of $g_{B^*B\pi}$, involving the finite-mass $b$-quarks [12] and presented in table 7. On the other hand, the result in (5.19) is within uncertainty consistent with our LCSR result. Finally, we also quote the earlier result $g_{B^*B\pi} = 30 \pm 5$ obtained in [58] using (5.17) together with the $z$-expansion of the $B \to \pi$ form factor calculated from LCSR. Since this calculation was done at small and intermediate momentum transfers, the extrapolation via $z$-expansion plays a more important role and the estimated uncertainty is naturally larger than for the lattice QCD results.

## 6 Conclusions and perspectives

In this paper we revisited the calculation of the strong couplings $g_{D^*D\pi}$ and $g_{B^*B\pi}$ from the LCSR that was originally derived in [13]. The method is based on the OPE of the correlation function (2.3) in terms of the pion DAs with growing twist. The use of a finite-mass heavy quark allows one to easily transform our sum-rule expressions to be applied for both charmed and bottom mesons.

Our main new result is the NLO twist-3 term in the LCSR, calculated from the gluon radiative corrections to the underlying correlation function. We also derived compact analytical expressions for both twist-2 and twist-3 NLO terms in a form of double dispersion relation. This derivation was done in the $\overline{\text{MS}}$ scheme for the heavy quark mass, and additional terms for a transition to the pole mass scheme were also obtained for the sake of generality. Among other new results, the continuum subtraction under the quark-hadron duality assumption is extended to all twist-3 and twist-4 terms at LO. We also carried out a detailed investigation of the sensitivity of LCSR to the form of two-dimensional duality region. For the dominant part of the spectral density which is concentrated near the diagonal on the plane of the two variables, the triangle region was found to be the most convenient choice. In addition, all input parameters entering LCSR were updated, with an emphasis on the key parameter — the midpoint value of the twist-2 pion DA.

As a result, the overall accuracy of the LCSRs for the strong couplings is substantially improved with respect to the earlier analyses in [13, 23]. Especially important, also numerically, is the inclusion of the new NLO twist-3 term in these sum rules. Due to a more precise input, the parametrical uncertainty for the default version of LCSRs — with the heavy-meson decay constants taken from the lattice QCD — is reduced to the level of 10%. Still, not completely included in this estimate is a "systematic uncertainty" caused by the quark-hadron duality which is more pronounced for the double dispersion relation, than for the sum rules based on the single-variable dispersion relation. One step in assessing this uncertainty was done here by examining the variation due to the shape of the duality region which was found to be rather small.

We compared our result for $g_{D^*D\pi}$ with the experimental measurement of this coupling inferred from the $D^* \to D\pi$ decay branching fraction and the $D^*$ total width. Identifying,

JHEP03(2021)016

somewhat qualitatively, as a $1\,\sigma$ standard deviation, the estimated uncertainty of $O(10\%)$ ($O(20\%)$) of the LCSR result for $g_{D^*D\pi}$ when the lattice-QCD values (two-point sum rules) are used for decay constants, we find that our result is smaller by approximately $2\,\sigma$ than (agrees within $1\,\sigma$ with) the measured value. The agreement is much better than before, when only the LO [13] or partial NLO [23] results were included in the numerical analysis. We conclude that there is no need for a radical modification of the quark-hadron duality ansatz, e.g. adding explicitly the radially excited heavy-meson states to the hadronic part of the sum rules, as suggested earlier [24, 25]. On the other hand, we found that the observed insignificant deviation of the LCSR prediction for $g_{D^*D\pi}$ from experiment can be removed by a moderate modification of the twist-2 pion DA at low scales. This modification, in its turn, yields quite a noticeable growth of our prediction for $g_{B^*B\pi}$. Hence, the sum rule for strong coupling considered here reveals a sensitivity to the pion twist-2 DA, similar to the other well-known LCSRs for the $\gamma^*\gamma \to \pi$ transition form factor, the pion e.m. form factor and $H \to \pi$ form factors ($H = D, B$).

Since there are no direct measurements of the $g_{B^*B\pi}$ coupling, we can only compare our result with the lattice QCD calculations. The most advanced calculation of [12] performed at a finite $b$-quark mass, however, neglecting the subleading power terms in the heavy quark expansion yields a $g_{B^*B\pi}$ coupling which is about 30% larger than the LCSR prediction (see table 7). On the other hand, as we have demonstrated, the latter is quite compatible with the value (5.19) extracted from the extrapolation to the $B^*$ pole of the lattice QCD results for the $B \to \pi$ form factor [61].

Finally, one of the main conclusions following from our analysis is that the inverse mass correction to the heavy-quark limit of the strong couplings is quite large. Hence, it is probably premature to compare our results for that limit with the effective coupling $\hat{g}$ inferred from the lattice QCD calculations performed in HQET. To clarify this issue, an alternative LCSR for the heavy-meson-pion strong coupling has to be derived in HQET and compared with the heavy-mass expansion of the sum rule considered here.

The method of LCSR considered in this paper is well suited to calculate a whole variety of strong couplings involving the pion. This can easily be done by varying the spin-parity or flavor quantum numbers of both interpolating currents in the correlation function. An early work in this direction can be found e.g. in [62] where the strong couplings of heavy mesons with other combinations of spin-parities were calculated. Switching to the strange quark in the interpolating currents allows one to access the strange counterparts of the strong couplings considered here, such as $g_{H_s^*HK}$ and $g_{H^*H_sK}$ for both $H = D, B$.

The limited scope of this paper prevents us from discussing in detail alternative sum rules for the strong $H^*H\pi$ couplings, e.g. the ones employing the external soft-pion field in which the correlation function of two heavy-light currents is expanded in terms of local operators (see [63–65]). A detailed derivation of this method and comparison with LCSR can be found in [13]. Due to the growing interest in the $B$-meson DAs, it will be also interesting to "invert" the correlation function considered in this paper to the one with the $B$-meson DAs and the pion-interpolating current.

JHEP03(2021)016

## Acknowledgments

First of all, we would like to thank Nils Offen and Patrick Gelhausen for participating at the early stages of this work. The work of A.K. is supported by the DFG (German Research Foundation) under grant 396021762-TRR 257 "Particle Physics Phenomenology after the Higgs Discovery". B.M. has been supported by the European Union through the European Regional Development Fund the Competitiveness and Cohesion Operational Programme (KK.01.1.1.06) and by the Croatian Science Foundation (HRZZ) project IP-2019-04-7094. B.M. would also like to express her gratitude to Wolfram Research for providing a free licence to use `Mathematica` at home during the COVID-19 pandemic lockdown and to Goran Duplančić for discussions. A.K. and B.M. are also partially supported by the Alexander von Humboldt Foundation in the framework of the Research Group Linkage Programme funded by the German Federal Ministry of Education and Research. Y.M.W. acknowledges support from the National Youth Thousand Talents Program, the Youth Hundred Academic Leaders Program of Nankai University, the National Natural Science Foundation of China with Grant No. 11675082, 11735010 and 12075125, and the Natural Science Foundation of Tianjin with Grant No. 19JCJQJC61100. Y.B.W. is supported in part by the Alexander-von-Humboldt Stiftung. We also would like to express a special thanks to the Mainz Institute for Theoretical Physics (MITP) of the Cluster of Excellence PRISMA+ (Project ID 39083149) for its hospitality and support during the scientific program "Light-cone distribution amplitudes in QCD" where this work was re-intiated.

## A Pion light-cone DAs

The definitions of the pion light-cone DAs in terms of the vacuum-to-pion matrix element of the quark-antiquark and quark-antiquark-gluon nonlocal operators can be found in [21]. Here the expressions for pion DAs used in the numerical analysis are collected:

- the twist-2 DA:

$$\varphi_\pi(u,\mu) = 6u\bar{u}\left[1 + \sum_{n=2,4,\dots} a_n(\mu) C_n^{3/2}(u-\bar{u})\right], \tag{A.1}$$

where $C_n^\alpha(z)$ are Gegenbauer polynomials and the scale dependence of the moments reads

$$a_n(\mu) = \left[\frac{\alpha_s(\mu)}{\alpha_s(\mu')}\right]^{\frac{\gamma_0^n}{\beta_0}} a_n(\mu'), \tag{A.2}$$

with $\beta_0 = 11 - \frac{2}{3}n_f$ and the anomalous dimension

$$\gamma_0^n = C_F\left[-3 - \frac{2}{(n+1)(n+2)} + 4\sum_{k=1}^{n+1}\left(\frac{1}{k}\right)\right]; \tag{A.3}$$

- the twist-3 two-particle DAs:

$$\phi^p_{3\pi}(u) = 1 + 30\frac{f_{3\pi}}{\mu_\pi f_\pi}C_2^{1/2}(u-\bar{u}) - 3\frac{f_{3\pi}\omega_{3\pi}}{\mu_\pi f_\pi}C_4^{1/2}(u-\bar{u})\,, \tag{A.4}$$

$$\phi^\sigma_{3\pi}(u) = 6u\bar{u}\left[1 + 5\frac{f_{3\pi}}{\mu_\pi f_\pi}\left(1 - \frac{\omega_{3\pi}}{10}\right)C_2^{3/2}(u-\bar{u})\right]\,; \tag{A.5}$$

- the twist-3 three-particle DA:

$$\Phi_{3\pi}(\alpha_i) = 360\alpha_1\alpha_2\alpha_3^2\left[1 + \frac{\omega_{3\pi}}{2}(7\alpha_3 - 3)\right]\,. \tag{A.6}$$

Transforming the integration variables, the initial expression for the contribution of this DA,

$$F^{\text{tw}3,\bar{q}Gq}(q^2,(p+q)^2) = 4m_Q f_{3\pi}\int_0^1 dv\int\mathcal{D}\alpha\frac{v(q\cdot p)\Phi_{3\pi}(\alpha_i)}{(m_Q^2-(q+(\alpha_1+\alpha_3 v)p)^2)^2}\,, \tag{A.7}$$

where $\mathcal{D}\alpha \equiv d\alpha_1 d\alpha_2 d\alpha_3\,\delta(1-\alpha_1-\alpha_2-\alpha_3)$, is reduced to (3.8) and we use the following notation:

$$\begin{aligned}\overline{\Phi}_{3\pi}(u) &\equiv \frac{1}{2u}\int\limits_0^u d\alpha_1(u-\alpha_1)\int\limits_{u-\alpha_1}^{1-\alpha_1}\frac{d\alpha_3}{\alpha_3^2}\Phi_{3\pi}(\alpha_1, 1-\alpha_1-\alpha_3, \alpha_3)\\ &= 5u^2\bar{u}^2\left[3 + \omega_{3\pi}\left(-1 + \frac{7}{4}u\right)\right]\,;\end{aligned} \tag{A.8}$$

- the twist-4 two-particle DAs:

$$\psi_{4\pi}(u) = \frac{20}{3}\delta_\pi^2 C_2^{1/2}(2u-1)\,, \tag{A.9}$$

with a shorthand notation for the integral in (3.9)

$$\bar{\psi}_{4\pi}(u) \equiv u\int_0^u dv\,\psi_{4\pi}(v) = \frac{20}{3}\delta_\pi^2 u^2\bar{u}(1-2u)\,, \tag{A.10}$$

and

$$\begin{aligned}\phi_{4\pi}(u) = \frac{200}{3}\delta_\pi^2 u^2\bar{u}^2 + 8\delta_\pi^2\epsilon_\pi\Big[&u\bar{u}(2+13u\bar{u}) + 2u^3(10-15u+6u^2)\ln(u)\\ &+ 2\bar{u}^3(10-15\bar{u}+6\bar{u}^2)\ln(\bar{u})\Big].\end{aligned} \tag{A.11}$$

- the twist-4 three-particle DAs:

$$\begin{aligned}\Phi_{4\pi}(\alpha_i) &= 120\delta_\pi^2\epsilon_\pi(\alpha_1-\alpha_2)\alpha_1\alpha_2\alpha_3,\\ \Psi_{4\pi}(\alpha_i) &= 30\delta_\pi^2(\alpha_1-\alpha_2)\alpha_3^2\left(\frac{1}{3} + 2\epsilon_\pi(1-2\alpha_3)\right),\\ \tilde{\Phi}_{4\pi}(\alpha_i) &= -120\delta_\pi^2\alpha_1\alpha_2\alpha_3\left(\frac{1}{3} + \epsilon_\pi(1-3\alpha_3)\right),\\ \tilde{\Psi}_{4\pi}(\alpha_i) &= 30\delta_\pi^2\alpha_3^2(1-\alpha_3)\left(\frac{1}{3} + 2\epsilon_\pi(1-2\alpha_3)\right).\end{aligned} \tag{A.12}$$

JHEP03(2021)016

Using these expressions, we transformed the sum of the contributions of the above DAs,

$$F^{\text{tw4},\bar{q}Gq}(q^2,(p+q)^2) = m_Q^2 f_\pi \int_0^1 dv \int \frac{\mathcal{D}\alpha}{(m_Q^2-(q+(\alpha_1+\alpha_3 v)p)^2)^2} \times \left[2\Psi_{4\pi}(\alpha_i)-\Phi_{4\pi}(\alpha_i)+2\tilde{\Psi}_{4\pi}(\alpha_i)-\tilde{\Phi}_{4\pi}(\alpha_i)\right]\Big|_{\alpha_2=1-\alpha_1-\alpha_3}, \tag{A.13}$$

to the compact form (3.11), denoting

$$\begin{aligned}\overline{\Phi}_{4\pi}(u) &= \int\limits_0^u d\alpha_1(u-\alpha_1)\int\limits_{u-\alpha_1}^{1-\alpha_1}\frac{d\alpha_3}{\alpha_3}\left[2\Psi_{4\pi}(\alpha_i)-\Phi_{4\pi}(\alpha_i)+2\tilde{\Psi}_{4\pi}(\alpha_i)-\tilde{\Phi}_{4\pi}(\alpha_i)\right]\Big|_{\alpha_2=1-\alpha_1-\alpha_3}\\ &= \frac{\delta_\pi^2}{3}u^3\bar{u}\left\{10-5u\left[1+3\epsilon_\pi\left(1-\frac{3}{5}u\right)\right]\right\}. \end{aligned}\tag{A.14}$$

## B Double spectral density at NLO

The expression for the twist-2 part of the NLO double spectral density in the sum rule (4.5) calculated in the $\overline{\text{MS}}$ scheme reads:

$$\begin{aligned}&\rho^{(\text{tw2,NLO})}(r_1,r_2)\\ &= f_\pi\,\theta(r_2-1)\Bigg\{6\Bigg[(r_1-1)(r_2-1)\left(3\ln\frac{m_Q^2}{\mu^2}+\ln^2\frac{r_1-1}{r_2-1}-\ln\frac{r_1-1}{r_2-1}\left(\ln\frac{r_1}{r_2}-\frac{1}{r_2}+1\right)\right.\\ &\quad\left.+\ln(r_2-1)\left[4-\ln(r_1r_2)-\frac{1}{r_2}\right]-\text{Li}_2(1-r_1)-3\,\text{Li}_2(1-r_2)+\frac{r_1}{r_2}-\frac{9}{2}-\frac{4}{3}\pi^2\right)\\ &\quad+(2-r_1-r_2)\left(3\ln\frac{m_Q^2}{\mu^2}-4\right)-r_2(r_1-1)\ln r_2-\frac{3}{2}(r_2-1)^2\Bigg]\frac{d^2}{d{r_1}^2}\delta(r_1-r_2)\\ &\quad+6\,\theta(r_1-1)(r_1-1)(r_2-1)\left[2\ln\frac{r_1-1}{r_2-1}-\ln\frac{r_1}{r_2}+\frac{1}{r_2}-1\right]\frac{d^3}{d{r_1}^3}\ln|r_1-r_2|\Bigg\},\end{aligned}\tag{B.1}$$

where we use the rescaled variables

$$r_1=\frac{s_1}{m_Q^2}\,,\qquad\text{and}\qquad r_2=\frac{s_2}{m_Q^2}\,. \tag{B.2}$$

To convert this result to the pole-mass scheme for the heavy quark, we are required to include the following term in the above expression

$$\begin{aligned}\Delta\rho^{(\text{tw2,NLO})}_{\text{pole}}(r_1,r_2) &= -6\,f_\pi\left(3\ln\frac{m_Q^2}{\mu^2}-4\right)\theta(r_2-1)\\ &\quad\times\left[(r_1-1)(r_2-1)+2-r_1-r_2\right]\frac{d^2}{d{r_1}^2}\delta(r_1-r_2)\,,\end{aligned}\tag{B.3}$$

with the replacement of $m_Q\to m_{Q,\,\text{pole}}$.

JHEP03(2021)016

The desired twist-3 spectral density at NLO in $\alpha_s$ is calculated here for the first time:

$$
\begin{aligned}
&\rho^{(\text{tw3,NLO})}(r_1,r_2)\\
&= f_\pi\,\frac{\mu_\pi}{m_Q}\Big\{\theta(r_2-1)\Big[-2\,(r_1+r_2)\left(3\,\ln\frac{m_Q^2}{\mu^2}-4\right)+4\,r_2\,(r_1-1)\,\ln\frac{r_2-1}{r_1-1}\\
&\quad+\frac{2\,r_1}{r_2}\,(r_2-1)^2\,\ln(r_2-1)-\Big[1+r_2+r_1\,(r_1+2)\,(r_2-1)\Big]\ln r_2+(r_1-1)\,(r_2-3)\\
&\quad+2\,(r_1+r_2-2r_1r_2)\Big(-\ln^2\frac{r_2-1}{r_1-1}-\ln(r_1-1)\ln\frac{r_2}{r_1}+2\ln(r_2-1)\ln r_2\\
&\quad+\frac{1}{r_2}\,\ln\frac{r_2-1}{r_1-1}+3\,\text{Li}_2(1-r_2)+\text{Li}_2(1-r_1)+\frac{4}{3}\,\pi^2\Big)\Big]\,\frac{d^2}{d{r_1}^2}\,\delta(r_1-r_2)\\
&\quad-2\,\theta(r_1-1)\,\theta(r_2-1)\,\Big[(r_1+r_2-2\,r_1\,r_2)\left(\ln\frac{r_2}{r_1}-2\,\ln\frac{r_2-1}{r_1-1}+\frac{1}{r_2}\right)\\
&\quad+2\,r_2\,(r_1-1)\Big]\,\frac{d^3}{d{r_1}^3}\,\ln|r_1-r_2|+4\left[\frac{d}{dr_1}\,\delta(r_1-1)\right]\delta(r_2-1)\left(3\,\ln\frac{m_Q^2}{\mu^2}-4\right)\Big\}\,. \qquad \text{(B.4)}
\end{aligned}
$$

The additional term needed to convert the above expression to the pole-mass scheme is

$$
\begin{aligned}
\Delta\rho^{(\text{tw3,NLO})}_{\text{pole}}(r_1,r_2) = \frac{f_\pi\,\mu_\pi}{m_Q}\left(3\,\ln\frac{m_Q^2}{\mu^2}-4\right)\Big\{&\theta(r_2-1)\,(3\,r_1+3\,r_2-2\,r_1\,r_2)\,\frac{d^2}{d{r_1}^2}\,\delta(r_1-r_2)\\
&-4\left[\frac{d}{dr_1}\,\delta(r_1-1)\right]\delta(r_2-1)\Big\}\,. \qquad \text{(B.5)}
\end{aligned}
$$

To facilitate the comparison of our results with those already derived in [23], we introduce the two dimensionless variables

$$
r=\frac{s_1-m_Q^2}{s_2-m_Q^2}\,,\qquad \sigma=\frac{s_1}{m_Q^2}+\frac{s_2}{m_Q^2}-2\,. \qquad \text{(B.6)}
$$

Taking into account the fact that the double spectral densities will be integrated over the variables $r$ and $\sigma$ (replacing $s_1$ and $s_2$) in the LCSR for the strong coupling $H^*H\pi$, the method of integration by parts (IBP) can be further applied to the $r$-variable integral in order to simplify the obtained lengthy expressions of (B.1) and (B.4) for the triangular duality region, which will result in the vanishing surface terms. Under this circumstance, the twist-2 NLO spectral density (B.1) can be cast into a more compact form

$$
\begin{aligned}
&\rho^{(\text{tw2,NLO})}(r,\sigma)\\
&= f_\pi\,\frac{(r+1)^2}{\sigma}\Big\{3\,\delta(r-1)\left[\delta(\sigma)-\frac{1}{2}\,\theta(\sigma)\right]\left(3\,\ln\frac{m_Q^2}{\mu^2}-4\right)+\theta(\sigma)\Big[\delta(r-1)\Big(2\,\pi^2\\
&\quad+6\,\text{Li}_2\left(-\frac{\sigma}{2}\right)+\ln\frac{\sigma}{2}\left[3\,\ln\frac{\sigma+2}{2}-\frac{3\,\sigma\,(\sigma+4)\,(3\,\sigma+10)+72}{2\,(\sigma+2)^3}\right]+\frac{3}{4}\,\frac{\sigma\,(\sigma+12)+12}{(\sigma+2)^2}\Big)\\
&\quad+\theta(r)\,\frac{6\,r}{(r+1)}\left(2\,\ln r+\ln\frac{1+r+\sigma}{1+r+r\,\sigma}-\frac{\sigma}{1+r+\sigma}\right)\frac{d^3}{dr^3}\,\ln|r-1|\Big]\Big\}\,. \qquad \text{(B.7)}
\end{aligned}
$$

JHEP03(2021)016

Switching to the pole-mass scheme of the heavy quark, one needs to add the following expression

$$\Delta\rho^{(\mathrm{tw2,NLO})}_{\mathrm{pole}}(r,\sigma) = f_\pi \,\frac{(r+1)^2}{\sigma}\left(3\,\ln\frac{m_Q^2}{\mu^2}-4\right)\left\{3\,\delta(r-1)\left[\frac{1}{2}\,\theta(\sigma)\,-\delta(\sigma)\right]\right\}. \tag{B.8}$$

It is straightforward to verify that the sum of (B.7) and (B.8) is equivalent to the expression obtained in [23] in the pole-mass scheme. Along the same vein, we express the twist-3 double spectral density at $\mathcal{O}(\alpha_s)$ (B.4) for the default duality region as follows

$$\begin{aligned}
&\rho^{(\mathrm{tw3,NLO})}(r,\sigma)\\
&= f_\pi\,\frac{\mu_\pi}{m_Q}\,\frac{(r+1)^2}{\sigma}\left\{\left(\delta(r-1)\left[\delta(\sigma)-2\,\delta'(\sigma)\right]+4\,(r+1)\,\delta(r)\,\delta'(\sigma)\right)\left(3\,\ln\frac{m_Q^2}{\mu^2}-4\right)\right.\\
&\quad+\left(\frac{4}{3}\pi^2+1\right)\delta(r-1)\,\delta(\sigma)+\theta(\sigma)\left[\delta(r-1)\left(\frac{4}{3}\pi^2+4\,\mathrm{Li}_2\left(-\frac{\sigma}{2}\right)+\frac{1}{2}\left(\frac{\sigma}{4}-\frac{4}{\sigma}\right)\ln\frac{\sigma+2}{2}\right.\right.\\
&\quad\left.+\ln\frac{\sigma}{2}\left(\frac{\sigma^2+4}{2\,(\sigma+2)^2}+2\,\ln\frac{\sigma+2}{2}\right)+\frac{1}{2\,(\sigma+2)^2}\left[\frac{3\,\sigma\left[\sigma\,(\sigma+12)+80\right]}{8}+\frac{32}{\sigma}+62\right]\right)\\
&\quad+\theta(r)\,\frac{2}{\sigma\,(r+1)}\left(\left[(1+r)^2+2\,r\,\sigma\right]\left(2\,\ln r+\ln\frac{1+r+\sigma}{1+r+r\,\sigma}\right)-\frac{(1+r)^2\,(r-1)}{1+r+\sigma}-2\,r\,\sigma\right)\\
&\quad\left.\left.\times\,\frac{d^3}{dr^3}\ln|r-1|\right]\right\},
\end{aligned} \tag{B.9}$$

and

$$\begin{aligned}
\Delta\rho^{(\mathrm{tw3,NLO})}_{\mathrm{pole}}(r,\sigma) &= f_\pi\,\frac{\mu_\pi}{m_Q}\,\frac{(r+1)^2}{\sigma}\left(3\,\ln\frac{m_Q^2}{\mu^2}-4\right)\\
&\quad\times\left\{\frac{1}{2}\,\delta(r-1)\Big[\theta(\sigma)+2\,\delta'(\sigma)-\,\delta(\sigma)\Big]-4\,(r+1)\,\delta(r)\,\delta'(\sigma)\right\}.
\end{aligned} \tag{B.10}$$

Finally, for completeness we present the pole-mass scheme additions to the final NLO expressions in (4.13), separately for the twist-2 and twist-3 parts:

$$\begin{aligned}
\Delta f^{(\mathrm{tw2})}_{\mathrm{pole}}(\sigma) &= \left(3\,\ln\frac{m_Q^2}{\mu^2}-4\right)3\left[\frac{1}{2}-\delta(\sigma-0^+)\right],\\
\Delta f^{(\mathrm{tw3})}_{\mathrm{pole}}(\sigma) &= \left(3\,\ln\frac{m_Q^2}{\mu^2}-4\right)\frac{1}{2}\Big[1-\delta(\sigma-0^+)-4\,\delta'(\sigma-0^+)\Big].
\end{aligned} \tag{B.11}$$

## C Two-point sum rules for heavy meson decay constants at NLO

Here the two-point QCD sum rules for the decay constants of the heavy-light mesons $H$ and $H^*$ ($H = D, B$) are presented. Their expressions — with the NLO, $O(\alpha_s)$ accuracy in the perturbative and quark-condensate terms and including the dimension $d \leq 6$ condensates — are taken from [28]. In these sum rules, we denote the Borel parameter squared and

JHEP03(2021)016

duality thresholds by the barred quantities $\bar{M}^2$ and $\bar{s}_0, \bar{s}_0^*$, to distinguish them from the analogous parameters in the LCSR for the strong coupling.

For the decay constant of the pseudoscalar meson we have:

$$f_H^2 = \frac{e^{m_H^2/\bar{M}^2}}{m_H^4}\left\{\int\limits_{m_Q^2}^{\bar{s}_0} ds\, e^{-s/\bar{M}^2}\rho_5^{(\text{pert})}(s) + \Pi_5^{\langle\bar{q}q\rangle}(\bar{M}^2) + \Pi_5^{(d456)}(\bar{M}^2)\right\}, \qquad \text{(C.1)}$$

where the perturbative spectral density (for a massless light quark) is:

$$\begin{aligned}\rho_5^{(\text{pert})}(s) = {} & \frac{3m_Q^2}{8\pi^2}s\,(1-z)^2 + \frac{3\alpha_s C_F}{16\pi^3}m_Q^2 s(1-z)\left[\frac{9}{2}(1-z)\right.\\ & + 2(1-z)\Big[2\,\text{Li}_2(z) + \ln z\ln(1-z)\Big] + (3-z)(1-2z)\ln z\\ & \left. -(1-z)(5-2z)\ln(1-z) + (1-3z)\left(3\ln\frac{\mu^2}{m_Q^2}+4\right)\right], \end{aligned}\qquad \text{(C.2)}$$

denoting $z = m_Q^2/s$. The quark condensate contribution is

$$\begin{aligned}\Pi_5^{\langle\bar{q}q\rangle}(\bar{M}^2) = {} & -m_Q^3\langle\bar{q}q\rangle e^{-\frac{m_Q^2}{\bar{M}^2}} \\ & \times\left\{1 - \frac{\alpha_s C_F}{2\pi}\left[\left(3\ln\frac{\mu^2}{m_Q^2}+4\right)\frac{m_Q^2}{\bar{M}^2} - 7 - 3\ln\frac{\mu^2}{m_Q^2} + 3\,\Gamma\!\left(0,\frac{m_Q^2}{\bar{M}^2}\right)e^{\frac{m_Q^2}{\bar{M}^2}}\right]\right\},\end{aligned}\qquad \text{(C.3)}$$

containing the incomplete gamma function $\Gamma(a,z) = \int_z^\infty t^{a-1}e^{-t}dt$.

The sum of contributions of the $d = 4,5,6$ (gluon, quark-gluon and four-quark) condensates is

$$\begin{aligned}\Pi_5^{(d456)}(\bar{M}^2) = {} & \left[\frac{\langle GG\rangle m_Q^2}{12} - \frac{m_0^2\langle\bar{q}q\rangle m_Q^3}{2\bar{M}^2}\left(1-\frac{m_Q^2}{2\bar{M}^2}\right)\right.\\ & \left. - \frac{16\pi r_{vac}\alpha_s\langle\bar{q}q\rangle^2 m_Q^2}{27\bar{M}^2}\left(1-\frac{m_Q^2}{4\bar{M}^2}-\frac{m_Q^4}{12\bar{M}^4}\right)\right]e^{-\frac{m_Q^2}{\bar{M}^2}}\ .\end{aligned}\qquad \text{(C.4)}$$

In the above equations, the following shorthand notations and parameterizations are used for the QCD vacuum condensate densities:

$$\langle 0|\bar{q}q|0\rangle \equiv \langle\bar{q}q\rangle\,, \qquad (\alpha_s/\pi)\langle 0|G^a_{\mu\nu}G^{a\,\mu\nu}|0\rangle \equiv \langle GG\rangle\,, \qquad \langle 0|g_s\bar{q}G^a_{\mu\nu}t^a\sigma^{\mu\nu}q|0\rangle = m_0^2\langle\bar{q}q\rangle\,,$$

and the four-quark condensate density, factorized [33] via vacuum insertion, is approximated by the square of quark condensates $r_{vac}\langle\bar{q}q\rangle^2$, where the numerical factor $r_{vac}$ parameterizes deviation from the factorization. Note that $m_0^2$ and the four-quark density multiplied by $\alpha_s$ are to a good precision scale-independent. The numerical values of all condensate parameters are presented in table 1.

The sum rule for the decay constant of the vector heavy-light meson is:

$$f_{H^*}^2 = \frac{e^{m_{H^*}^2/\bar{M}^2}}{m_{H^*}^2}\left[\int\limits_{m_Q^2}^{\bar{s}_0^*} ds\, e^{-s/\bar{M}^2}\rho_T^{(\text{pert})}(s) + \Pi_T^{\langle\bar{q}q\rangle}(M^2) + \Pi_T^{(d456)}(M^2)\right], \qquad \text{(C.5)}$$

JHEP03(2021)016

with the perturbative spectral density

$$\rho_T^{(\text{pert})}(s) = \frac{1}{8\pi^2} s\,(1-z)^2\,(2+z) + \frac{3\alpha_s C_F}{16\pi^3} s\Bigg[1 - \frac{5}{2}z + \frac{2}{3}z^2 + \frac{5}{6}z^3 + \frac{2}{3}(1-z)^2(2+z)\Big[2\,\text{Li}_2(z) + \ln z \ln(1-z)\Big] + \frac{1}{3}z(-5-4z+5z^2)\ln z - \frac{1}{3}(1-z)^2(4+5z)\ln(1-z) - z(1-z^2)\left(3\ln\frac{\mu^2}{m_Q^2}+4\right)\Bigg], \tag{C.6}$$

and the condensate contributions:

$$\Pi_T^{\langle\bar{q}q\rangle}(\bar{M}^2) = -m_Q\langle\bar{q}q\rangle e^{-\frac{m_Q^2}{\bar{M}^2}}\left\{1 + \frac{\alpha_s C_F}{2\pi}\left[1 - 3\frac{m_Q^2}{\bar{M}^2}\ln\frac{\mu^2}{m_Q^2} - 4\frac{m_Q^2}{\bar{M}^2} + \frac{m_Q^2}{\bar{M}^2}e^{\frac{m_Q^2}{\bar{M}^2}}\Gamma\left(-1,\frac{m_Q^2}{\bar{M}^2}\right)\right]\right\}, \tag{C.7}$$

$$\Pi_T^{(d456)}(\bar{M}^2) = \left[-\frac{\langle GG\rangle}{12} + \frac{m_0^2\langle\bar{q}q\rangle m_Q^3}{4\bar{M}^4} - \frac{32\pi\alpha_s r_{vac}\langle\bar{q}q\rangle^2}{81\bar{M}^2}\left(1+\frac{m_Q^2}{\bar{M}^2}-\frac{m_Q^4}{8\bar{M}^4}\right)\right]e^{-\frac{m_Q^2}{\bar{M}^2}}. \tag{C.8}$$

**Open Access.** This article is distributed under the terms of the Creative Commons Attribution License (CC-BY 4.0), which permits any use, distribution and reproduction in any medium, provided the original author(s) and source are credited.
